# Supplementary material for: Assessment of Biological Activity of Low Molecular Weight 1,4-Benzoquinone Derivatives
Source: Biomolecules. 2025 Aug 14;15(8):1162. doi: 10.3390/biom15081162 (PMC12384008; doi:10.3390/biom15081162)
Supplement: Supplementary file 1 [file biomolecules-15-01162-s001.zip › biomolecules-3715784-supplementary.pdf]

Assessment of Biological Activity of Low Molecular Weight 1,4-Benzoquinone Derivatives

Marija Bartolić [1], Ana Matošević [1], Nikola Maraković [1], Irena Novaković [2], Dušan Sladić [3], Suzana Žunec [1], Dejan Opsenica [2,4] and Anita Bosak [1,*]

[1] Institute for Medical Research and Occupational Health, Ksaverska cesta 2, 10001 Zagreb, Croatia; mbartolic@imi.hr (M.B.); amatosevic@imi.hr (A.M.); nmarakovic@imi.hr (N.M.); suzana@imi.hr (S.Ž.)
[2] Institute of Chemistry Technology and Metallurgy, University of Belgrade, Njegoševa 12, 11000 Belgrade, Serbia; irena.novakovic@ihtm.bg.ac.rs (I.N.); dejan.opsenica@ihtm.bg.ac.rs (D.O.)
[3] Faculty of Chemistry, University of Belgrade, Studentski trg 12-16, 11158 Belgrade, Serbia; dsladic@chem.bg.ac.rs
[4] Centre of Excellence in Environmental Chemistry and Engineering, Njegoševa 12, 11000 Belgrade, Serbia
* Correspondence: abosak@imi.hr

Table of contents

1. Synthesis S2
1.1. General information S2
1.2. Synthetic procedures S2
1.3. ^{1}H and ^{13}C NMR spectra of tested compounds S6
1.4. (+) ESI-MS spectra of the tested compounds S15
2. 2D view of interactions
2.1. AChE and selected ligands S24
2.2. BChE and selected ligands S25
3. Calculated physical-chemical parameters of tested 1,4-benzoquinones S26
4. Human intestinal absorption S27

1. Synthesis

1.1. General information

Reagents and solvents were obtained from commercial sources (Fluka, Sigma, Aldrich, Merck or Acros Organics). Solvents were distilled before use, while the other chemicals were used as received. The reactions were monitored by Supelco TLC aluminium sheets, Silica gel 60 with UV indicator (254 nm) for chromatogram visualisation. Preparative thin-layer chromatography (TLC) was performed on Supelco silica gel 60 GF254 with a UV-active indicator and appropriate mobile phase indicated in the corresponding synthetic procedure. The yields refer to purified products. NMR spectra were recorded in deuterochloroform on a Bruker Avance III (500 MHz instrument for ^{1}H NMR and 125 MHz for ^{13}C NMR). Chemical shifts are reported in parts per million (ppm) using tetramethylsilane as the internal standard. The coupling constants (J) are given in hertz (Hz), and the multiplets are designated as follows: s, singlet; bs, broad singlet; d, doublet; dd, doublet of doublets; t, triplet; m, multiplet. ESI-MS spectra of the synthesized compounds were recorded on an Agilent Technologies 1200 Series instrument equipped with a Zorbax Eclipse Plus C18 (100 × 2.1 mm i.d., 1.8 μm) column and a DAD detector (190-450 nm) in combination with an Agilent Technologies 6210 Time-Of-Flight LC-MS instrument in positive ion mode with CH3CN/H2O 1/1 with 0.2 % HCOOH as the carrying solvent solution. Samples were dissolved in MeOH (HPLC grade purity). The capillary voltage = 4 kV, gas temperature = 350 °C, drying gas flow rate = 12 L min−1, nebulizer pressure = 45 psi and fragmentor voltage = 70 V were used.

Since the 1,4-benzoquinones studied are also conjugated carbonyl compounds that readily undergo nucleophilic attack, especially in the presence of acid, which is usually present in reversed-phase conditions, purities of the examined compounds are based on NMR spectra and HRMS data, along with accompanying chromatograms obtained during analysis. For all compounds, the purity is greater than 95%.

1.2. Synthetic procedures

New derivatives were synthetized starting from *tert*-butyl-1,4-benzoquinone and the corresponding thiol, in 96% ethanol and saturated aqueous solution $NaHCO_3$ under inert atmosphere (Scheme S1). Synthesis, analytical and spectral data of compounds **10**[31]**, 11,**[30, 63] **12,**[30] **13,**[30] and **14**,[32, 33] were published previously.

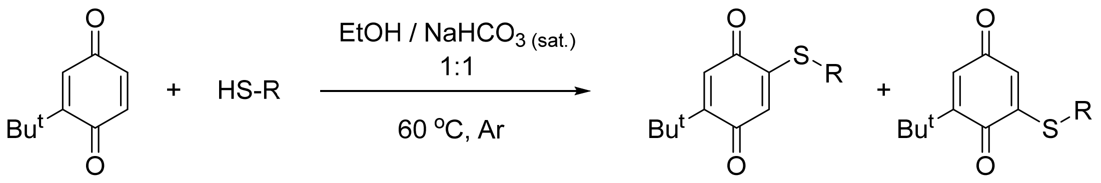

1

Scheme S1. Synthesis of thio-derivatives of *tert*-butyl-1,4-benzoquinone

***tert*-Butyl-1,4-benzoquinone (1)** was synthesized according to the procedure described in ref 3.

General procedure for the synthesis of alkyl/arylthio derivatives. Solution of *tert*-butyl-1,4-benzoquinone (328.4 mg, 2.0 mmol, 2 eq) and corresponding thiol (1 eq) was stirred in 40 mL of a mixture of 96% ethanol and saturated aqueous solution $NaHCO_3$ (1:1, V/V), under an argon atmosphere, at 60 °C for 3 hours, and then stirring was continued at room temperature overnight. Ethanol was removed by distillation under reduced pressure, and the product was isolated after extraction with dichloromethane (4 × 15 mL). Organic layers were washed with brine, dried over anhydrous sodium-sulphate, the solvent was removed under reduced pressure, and products were isolated using preparative thin-layer chromatography on silica gel.

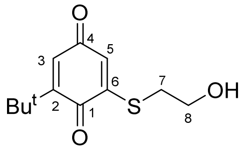

2-*tert*-Butyl-6-((2-hydroxyethyl)thio)-1,4-benzoquinone (2):

Preparative TLC: silica gel 60 GF254, mobile phase dichloromethane : methanol (96 / 4). Pale yellow oil, yield 28.8 mg (13%). ^{1}H NMR (500 MHz, $CDCl_3$): 1.29 (s, 9H, Bu^t), 3.01 (t, 2H, J = 6.1, H-C(7)), 3.93 (t, 2H, J = 6.1, H-C8)), 6.41 (d, 1H, J = 2.4, H-C(5)), 6.56 (d, 1H, J = 2.4 H-C(5)). ^{13}C NMR (125 MHz, $CDCl_3$): 29.33 (3 x Me), 33.53 (C-$(CH_3)_3$), 35.71 (C7), 59.87 (C8), 124.55 (C5), 132.62 (C3), 154.08 (C6), 156.17 (C2), 184.02 (C1), 185.03 (C4). (+)ESI-MS $[C_{12}H_{16}O_3S + H]^+$, calculated m/z 241.0893; found 241.0901.

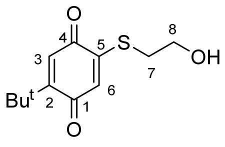

2-*tert*-Butyl-5-((2-hydroxyethyl)thio)-1,4-benzoquinone (3):

Preparative TLC: silica gel 60 GF254, mobile phase dichloromethane : methanol (96 / 4). Yellow oil, yield 19.2 mg (8%). ^{1}H NMR (500 MHz, CDCl$_3$): 1.31 (s, 9H, Bu^t), 3.03 (t, 2H, J = 6.1, H-C(7)), 3.93 (t, 2H, J = 6.1, H-C8)), 6.40 (s, 1H, H-C(6)), 6.66 (s, 1H, H-C(3)). ^{13}C NMR (125 MHz, CDCl$_3$): 29.48 (3 x Me), 33.08 (*C*-(CH$_3$)$_3$), 35.65 (C7), 59.98 (C8), 127.74 (C6), 131.53 (C3), 150.00 (C5), 157.35 (C2), 184.43 (C4), 185.04 (C1). (+)ESI-MS [$C_{12}H_{16}O_3S$ + H]$^+$, calculated m/z 241.0893; found 241.0905.

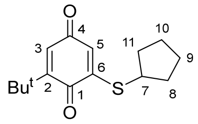

2-tert-Butyl-6-((cyclopentyl)thio)-1,4-benzoquinone (4):

Preparative TLC: silica gel 60 GF254, mobile phase dichloromethane.Yellow oil, yield 47.5 mg (18%). ^{1}H NMR (500 MHz, CDCl$_3$): 1.25 (s, 9H, Bu^t), 1.56–1.72 (m, 4H, H-(C9) and H-(C10)), 1.72–1.83 (m, 2H, Hax-(C8) and Hax-(C11)), 2.05–2.24 (m, 2H, Heq-(C8) and Heq-C(11)), 3.45–3.32 (m, 1H, H-C(7)), 6.38 (d, 1H, J = 2.4, H-C(5)), 6.50 (d, 1H, J = 2.4, H-C(3)). ^{13}C NMR (125 MHz, CDCl$_3$): 25.25 (C9 and C10), 29.33 (3 x Me), 33.02 (C8 and C11), 35.63 (*C*-(CH$_3$)$_3$), 42.84 (C7), 124.90 (C5), 132.63 (C3), 155.52 (C6), 155.96 (C2), 184.34 (C1), 185.12 (C4). (+)ESI-MS [$C_{15}H_{20}O_2S$ + H]$^+$, calculated *m/z* 265.1257; found 265.1276.

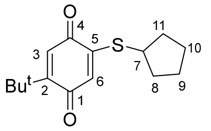

2-*tert*-Butyl-5-((cyclopentyl)thio)-1,4-benzoquinone (5):

Preparative TLC: silica gel 60 GF254, mobile phase dichloromethane. Pale yellow oil, yield 87.2 mg (33%). ^{1}H NMR (500 MHz, CDCl$_3$) 1.24 (s, 9H, Bu^t), 1.56–1.70 (m, 4H, H-(C9) and H-(C10)), 1.70–1.82 (m, 2H, Hax-(C8) and Hax-(C11)), 2.05–2.20 (m, 2H, Heq-(C8) and Heq-C(11)), 3.32-3.43 (m, 1H, H-C(7)), 6.31 (s, 1H, H-C(6)), 6.56 (s, 1H, H-C(3)). ^{13}C NMR (125 MHz, CDCl$_3$), 25.17 (C9 and C10), 29.44 (3 x Me), 33.03 (C8 and C11), 35.53 (*C*-(CH$_3$)$_3$), 42.40 (C7), 127.90 (C6), 131.38 (C3), 151.43 (C5), 157.11 (C2), 184.43 (C1), 185.36 (C4). (+)ESI-MS [$C_{15}H_{20}O_2S$ + H]$^+$, calculated *m/z* 265.1257; found 265.1273.

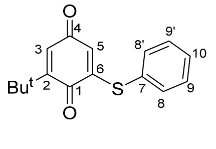

2-*tert*-Butyl-6-(phenylthio)-1,4-benzoquinone (6):

Preparative TLC: silica gel 60 GF254, mobile phase toluene : ethyl acetate (9 / 1). Pale yellow oil, yield 82.4 mg (30.3%). ^{1}H NMR (500 MHz, CDCl$_3$): 1.22 (s, 9H, Bu^t), 5.73 (d, 1H, J = 2.4, H-C(5)), 6.41 (d, 1H, J = 2.4, H-C(3)), 7.33–7.47 (m, 5H, Ar). ^{13}C NMR (125 MHz, CDCl$_3$): 29.10 (3 x Me), 35.34 (*C*-

($(CH_3)_3$), 124.96 (C10), 127.64 (C5), 130.25 (C3), 130.39 (C9 and C9’), 132.38 (C7), 135.60 (C8 and C8’), 155.26 (C2), 155.28 (C6), 183.87 (C1), 185.04 (C4). (+)ESI-MS $[C_{16}H_{16}O_2S + H]^+$, calculated *m/z* 273.0944; found 273.0948.

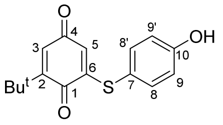

2-*tert*-Butyl)-6-((4-hydroxyphenyl)thio)-1,4-benzoquinone (7):

Preparative TLC: silica gel 60 GF254, mobile phase toluene : ethyl acetate (1 / 1). Brown oil, yield 34.6 mg (12%). ^{1}H NMR (500 MHz, $CDCl_3$): 1.28 (s, 9H, Bu^t), 5.81 (d, 1H, J = 2.4, H-C(5)), 6.49 (d, 1H, J = 2.4, H-C(3)), 6.69 (s, 1H, OH), 6.86 (d, 2H, J = 8.5, H-(C9) and H-C(9’)), 7.28 (d, 2H, J = 8.5, H-C(8) and H-(C8’)). ^{13}C NMR (125 MHz, $CDCl_3$): 29.11 (3 x Me), 35.47 (*C*-$(CH_3)_3$), 117.16 (C9 and C9’), 117.41 (C7), 124.49 (C5), 132.40 (C3), 137.15 (C8 and C8’), 156.11 (C10), 158.16 (C6), 158.13 (C2), 184.02 (C1), 185.97 (C4). (+)ESI-MS $[C_{16}H_{16}O_3S + H]^+$, calculated *m/z* 289.0893; found 289.0893.

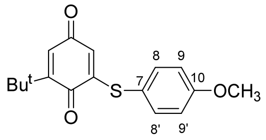

2-*tert*-Butyl-6-((4-methoxyphenyl)thio)-1,4-benzoquinone (8):

Preparative TLC: silica gel 60 GF254, mobile phase chloroform. Yellow oil, yield 111.8 mg (37%). ^{1}H NMR (500 MHz, $CDCl_3$): 1.26 (s, 9H, Bu^t), 3.81 (s, 3H, CH_3-O), 5.76 (d, 1H, J = 2.4 H-C(5)), 6.44 (d, 1H, J = 2.4, H-C(3)), 6.91–6.99 (m, 2H, H-C(9) and H-C(9’)), 7.31–7.38 (m, 2H, H-C(8) and H-C(8’)). ^{13}C NMR (125 MHz, $CDCl_3$): 29.24 (3 x Me), 35.49 (*C*-$(CH_3)_3$), 55.56 (CH_3-O), 116.02 (C9 and C9’), 117.93 (C7), 125.08 (C5), 132.52 (C3), 137.19 (C8 and C8’), 155.58 (C6), 157.29 (C2), 161.49 (C10), 184.29 (C4), 185.38 (C1). (+)ESI-MS $[C_{17}H_{18}O_3S + H]^+$, calculated *m/z* 303.1049; found 303.1036.

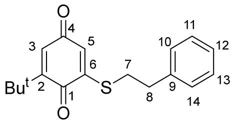

2-*tert*-Butyl-6-(phenethylthio)-1,4-benzoquinone (9):

Preparative TLC: silica gel 60 GF254, mobile phase chloroform. Pale brown oil, yield 33.0 mg (11%). ^{1}H NMR (500 MHz, $CDCl_3$) 1.25 (s, 9H, Bu^t), 3.05–2.85 (m, 4H, H-(C7) and H-(C8)), 6.32 (d, 1H, J = 2.3, H-C(5)), 6.51 (d, 1H, J = 2.3, H-C(3)), 7.15–7.25 (m, 3H, H-(C11), H-(C12) and H-(C14)), 7.26–7.33 (m, 2H, H-(C10) and H-(C14)). ^{13}C NMR (125 MHz, $CDCl_3$), 29.08 (3 x Me), 32.13 (C7), 33.64 (*C*-$(CH_3)_3$), 35.40 (C8), 126.90 (C11), 123.93 (C12), 126.90 (C11 and C13), 128.41 (C10 and C14), 128.73 (C5), 132.38 (C3), 139.00 (C9), 154.47 (C6), 155.71 (C2), 183.76 (C1), 184.68 (C4). (+)ESI-MS $[C_{18}H_{20}O_2S + H]^+$, calculated *m/z* 301.1257; found 301.1258.

2-*tert*-Butyl-6-(naphthalen-2-ylthio)-1,4-benzoquinone (10): 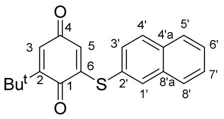

Preparative TLC: silica gel 60 GF254, mobile phase dichloromethane. Brown oil, yield 32.2 mg (10%). ^{1}H NMR (500 MHz, $CDCl_3$): 1.30 (s, 9H, Bu^{t}), 5.80 (d, 1H, *J* = 2.4, H-C(5)), 6.47 (d, 1H, *J* = 2.4, H-C(3)), 7.42–7.47 (m, 1H, H-C3')), 7.55 (m, 2H, H-C(6') and H-C(7')), 7.78 - 7.92 (m, 2H, H-(C4') and H-C(5')), 8.3 (s, 1H, H-(C1')). ^{13}C NMR (125 MHz, $CDCl_3$): 29.13 (3 x Me), 35.41 (*C*-$(CH_3)_3$), 124.73 (C6'), 125.22 (C7'), 127.04 (C3'), 127.81 (C5'), 127.87 (C8'), 127.90 (C5), 130.13 (C4'), 130.97 (C2'), 132.42 (C4'a), 133.64 (C3), 133.91 (C1'), 136.05 (C8'a), 156.36 (C6), 155.48 (C2), 184.00 (C1), 185.15 (C4). (+)ESI-MS $[C_{20}H_{18}O_2S + H]^+$, calculated *m/z* 323.1100; found 323.1096.

1.3. 1H and 13C NMR spectra of tested compounds

^{1}H NMR spectrum of 2-*tert*-butyl-6-((2-hydroxyethyl)thio)-1,4-benzoquinone (**2**)

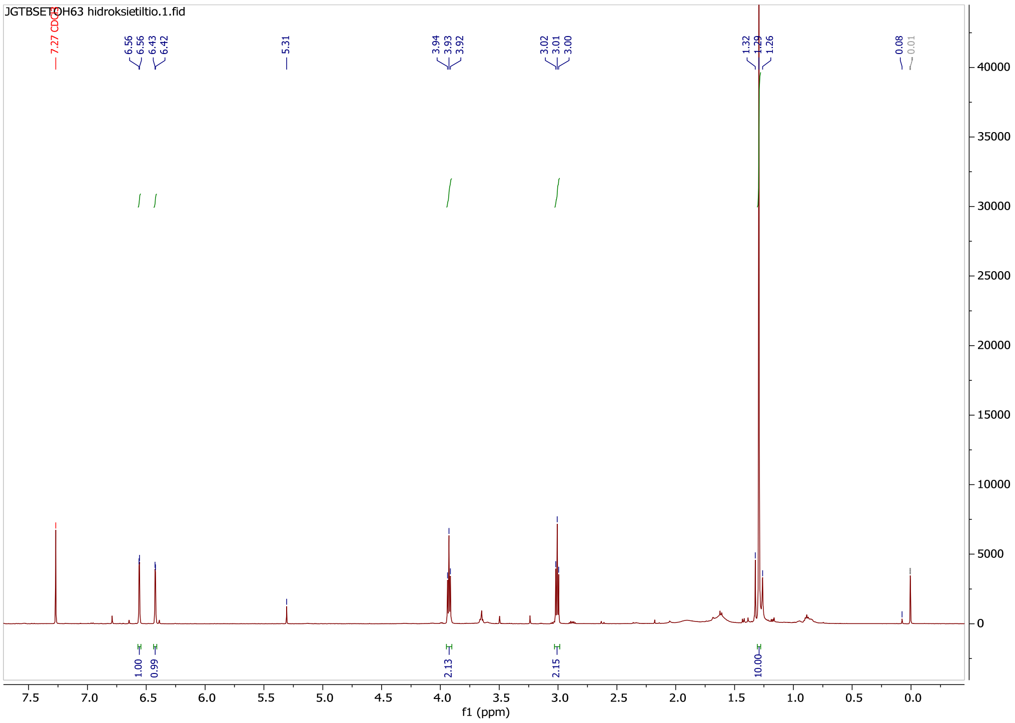

^{13}C NMR spectrum of 2-*tert*-butyl-6-((2-hydroxyethyl)thio)-1,4-benzoquinone (**2**)

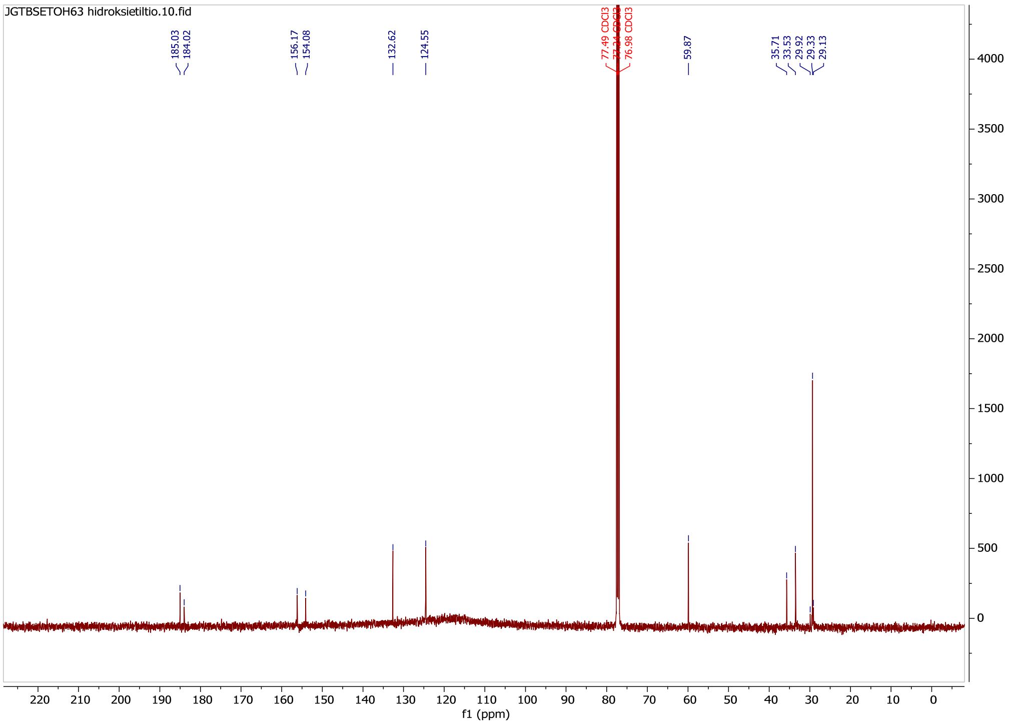

^{1}H NMR spectrum of 2-*tert*-butyl-5-((2-hydroxyethyl)thio)-1,4-benzoquinone (**3**)

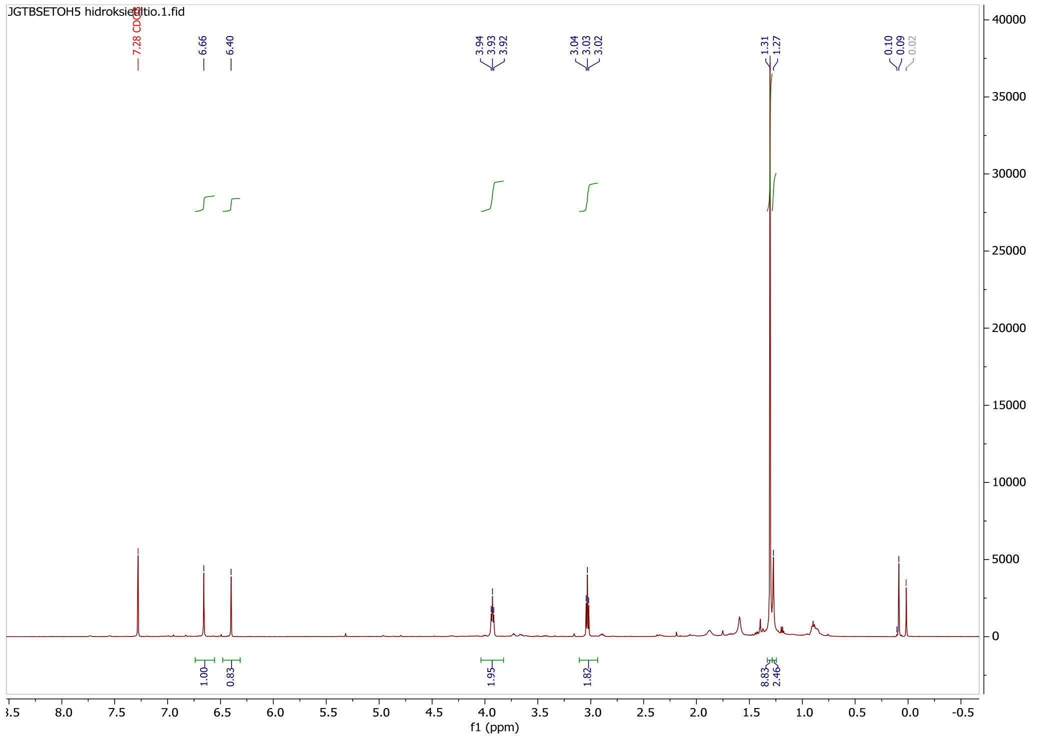

^{13}C NMR spectrum of 2-*tert*-butyl-5-((2-hydroxyethyl)thio)-1,4-benzoquinone (**3**)

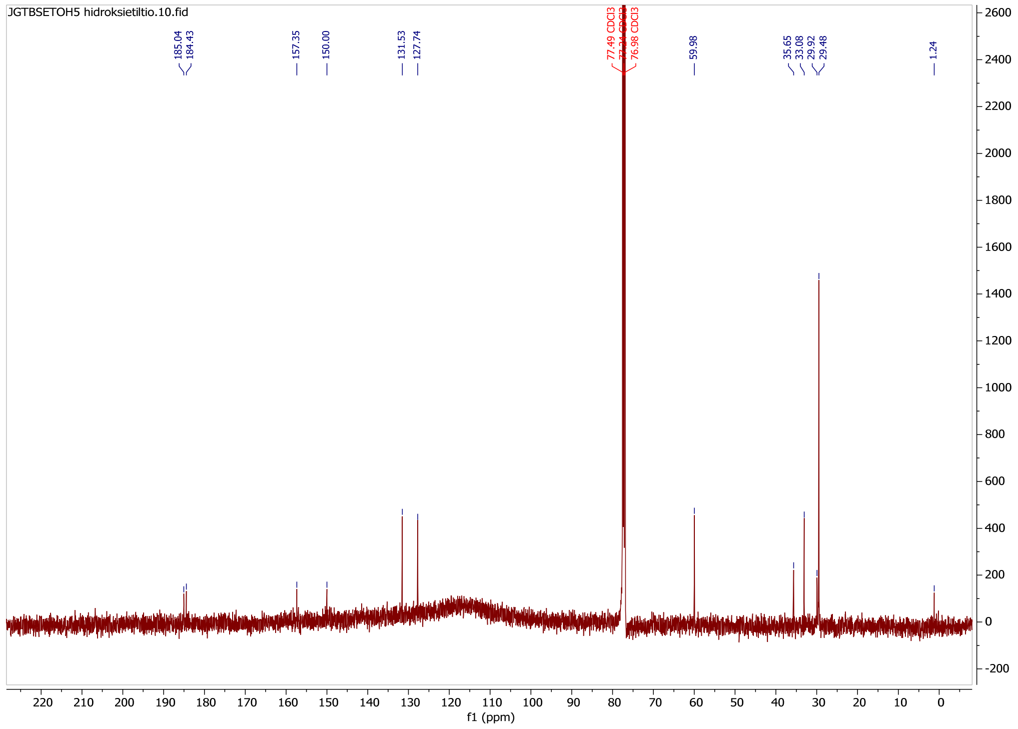

^{1}H NMR spectrum of 2-*tert*-butyl-6-(cyclopentylthio)-1,4-benzoquinone (**4**)

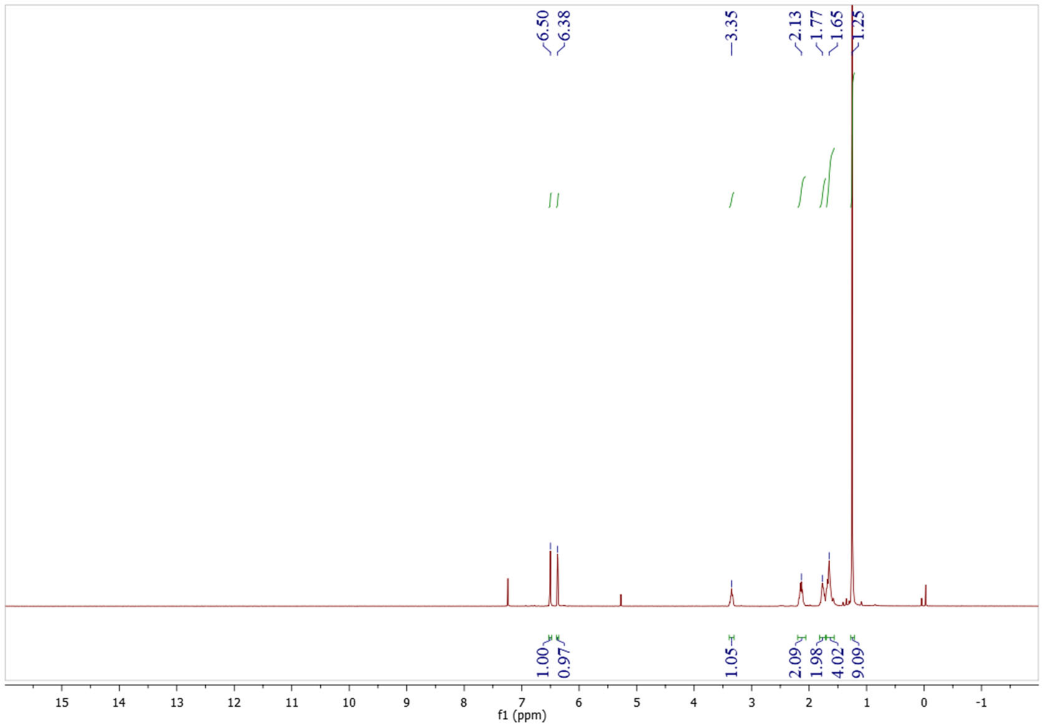

^{13}C NMR spectrum of 2-*tert*-butyl-6-(cyclopentylthio)-1,4-benzoquinone (**4**)

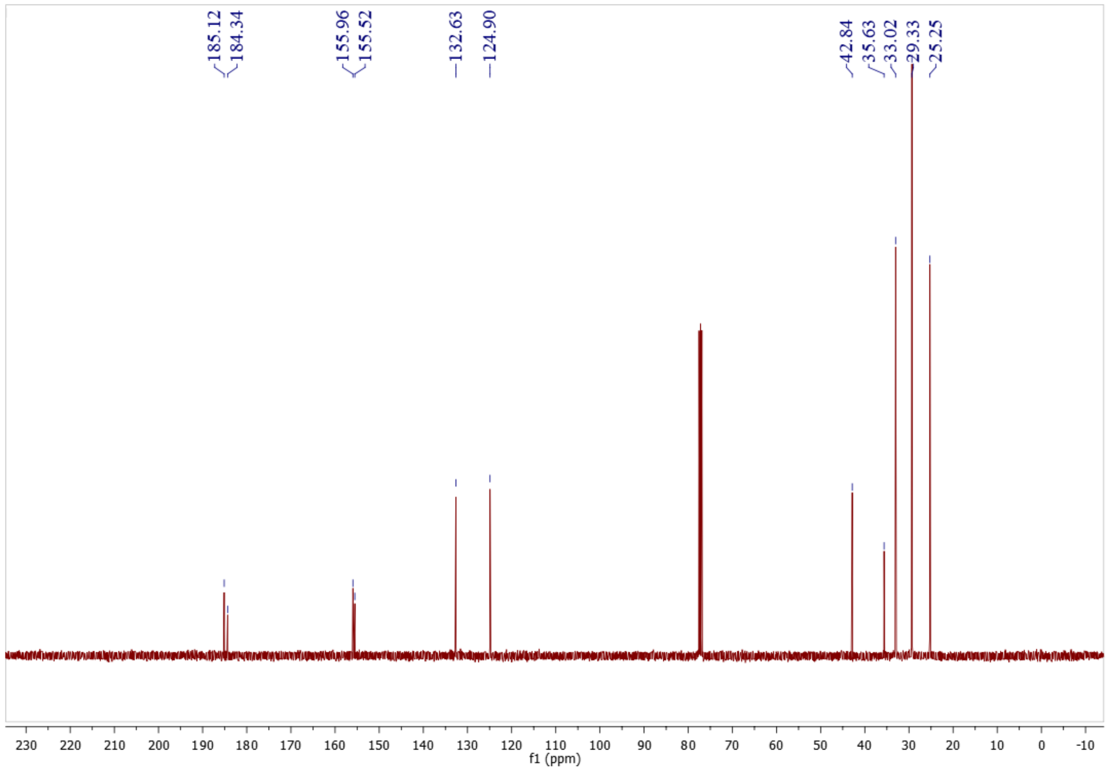

^{1}H NMR spectrum of 2-*tert*-butyl-5-(cyclopentylthio)-1,4-benzoquinone (**5**)

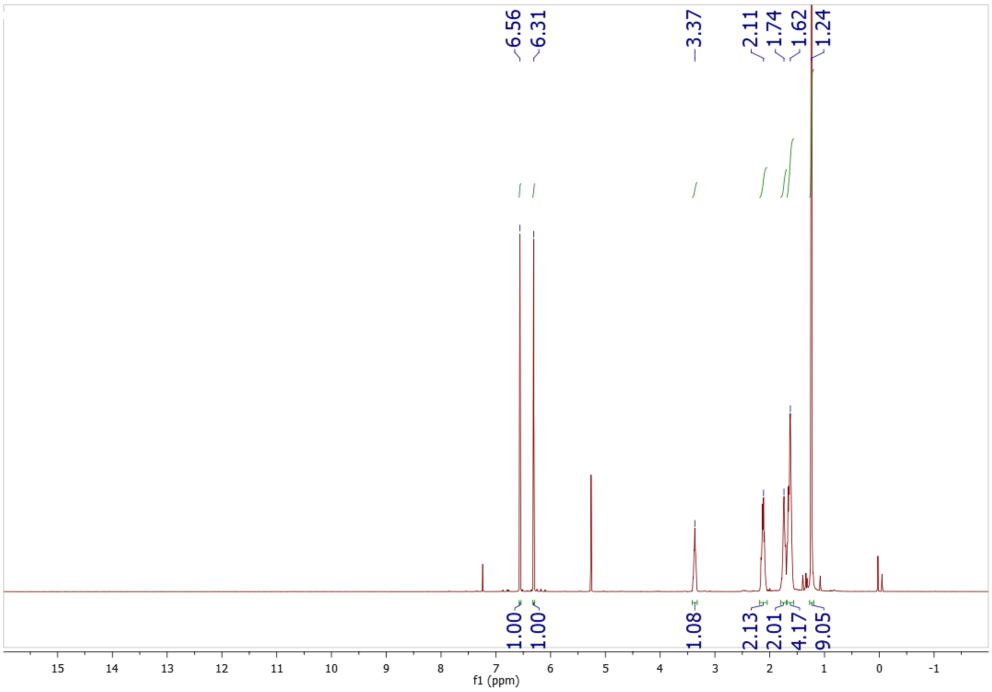

^{13}C NMR spectrum of 2-*tert*-butyl-5-(cyclopentylthio)-1,4-benzoquinone (**5**)

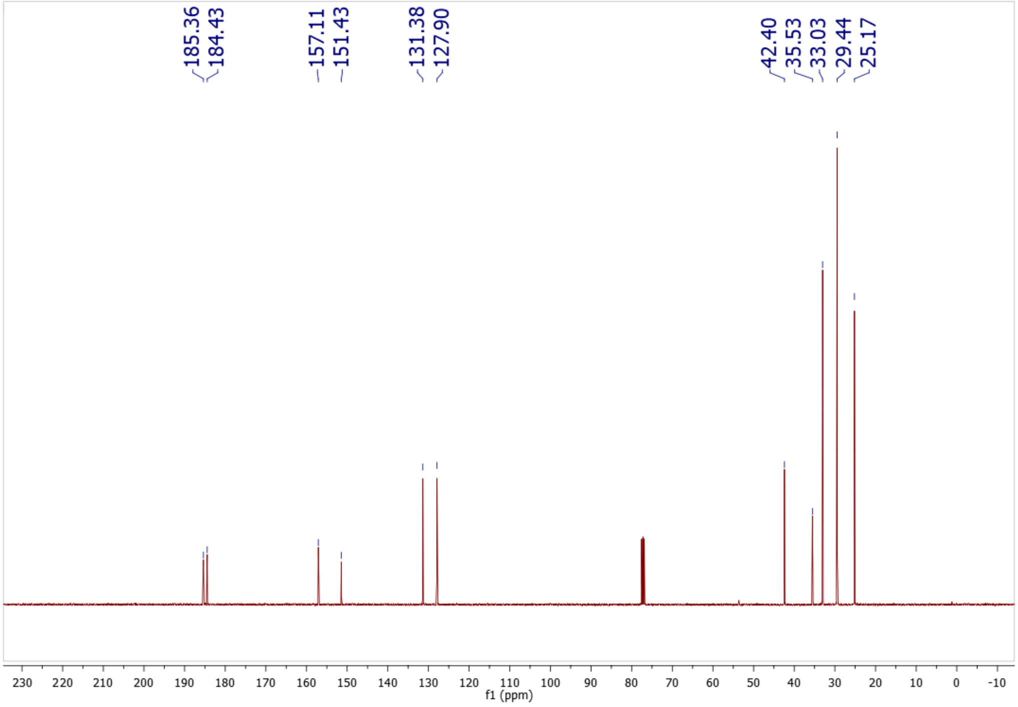

^{1}H NMR spectrum of 2-*tert*-butyl-6-(phenylthio)-1,4-benzoquinone (**6**)

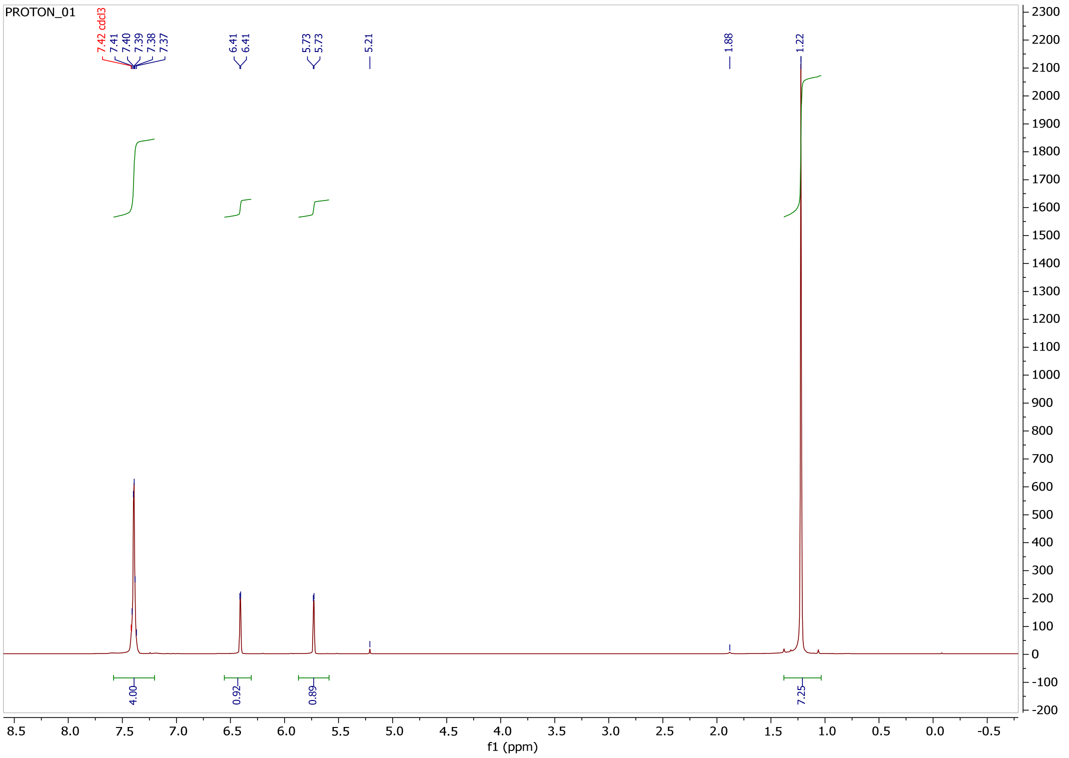

^{13}C NMR spectrum of 2-*tert*-butyl-6-(phenylthio)-1,4-benzoquinone (**6**)

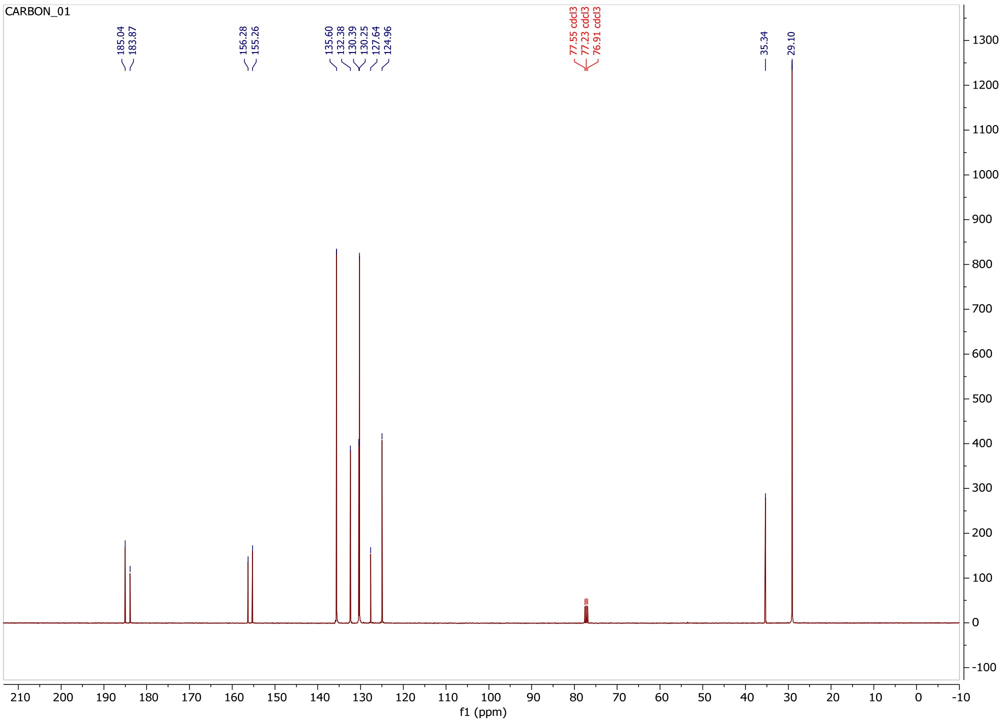

^{1}H NMR spectrum of 2-*tert*-butyl-6-((4-hydroxyphenyl)thio)-1,4-benzoquinone (**7**)

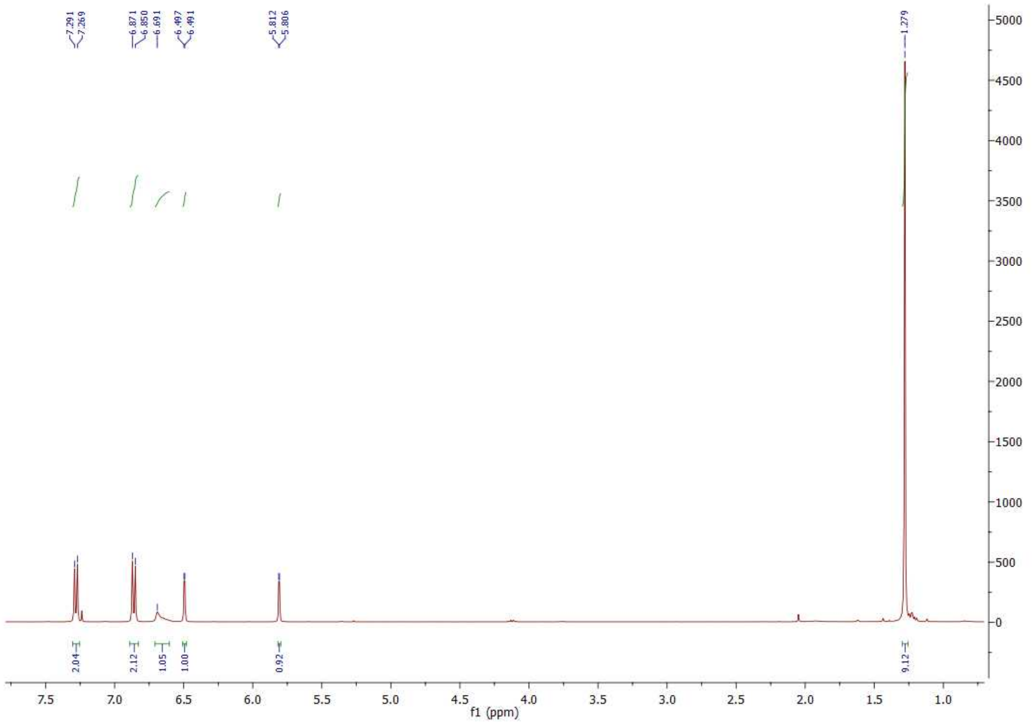

^{13}C NMR spectrum of 2-*tert*-butyl-6-((4-hydroxyphenyl)thio)-1,4-benzoquinone (**7**)

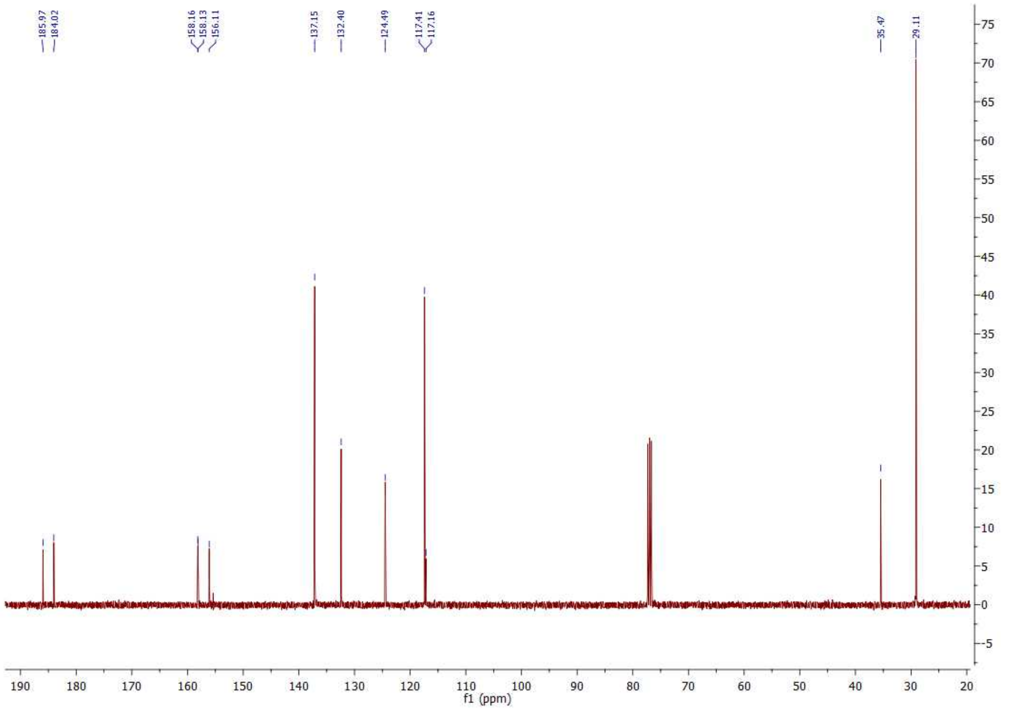

^{1}H NMR spectrum of 2-*tert*-butyl-6-((4-methoxyphenyl)thio)-1,4-benzoquinone (**8**)

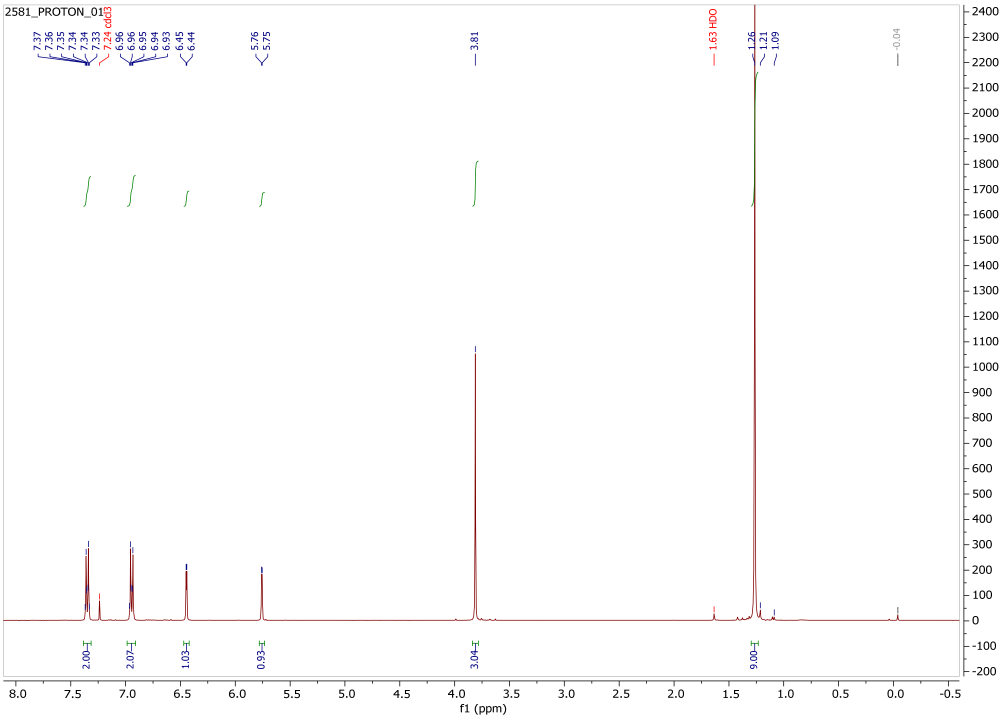

^{13}C NMR spectrum of 2-*tert*-butyl-6-((4-methoxyphenyl)thio)-1,4-benzoquinone (**8**)

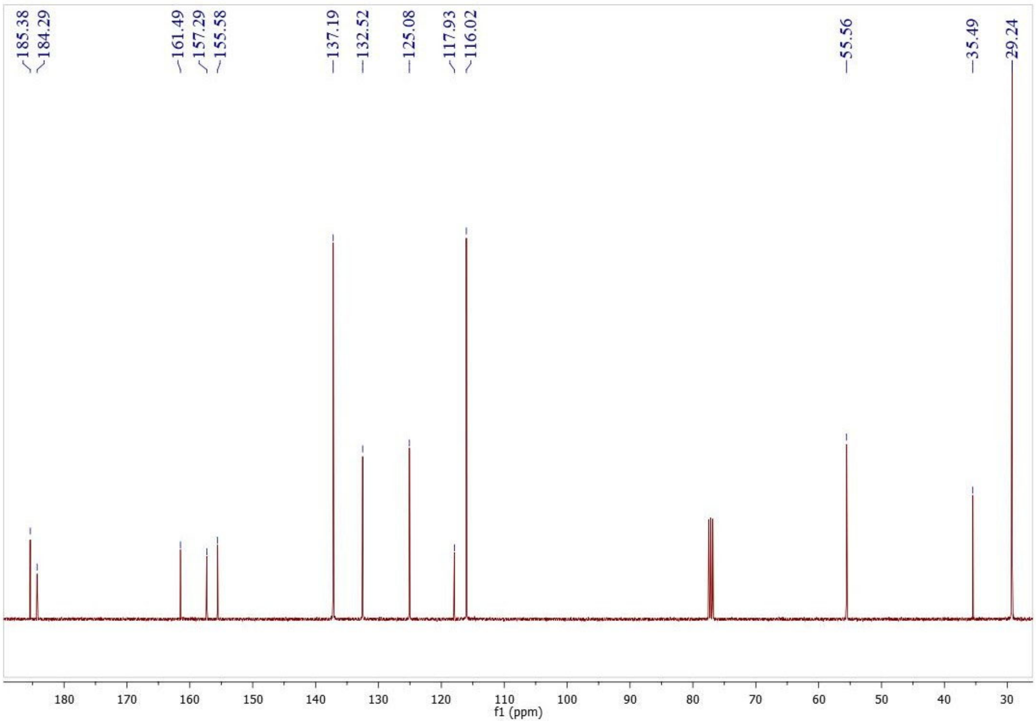

^{1}H NMR spectrum of 2-*tert*-butyl-6-(phenethylthio)-1,4-benzoquinone (**9**)

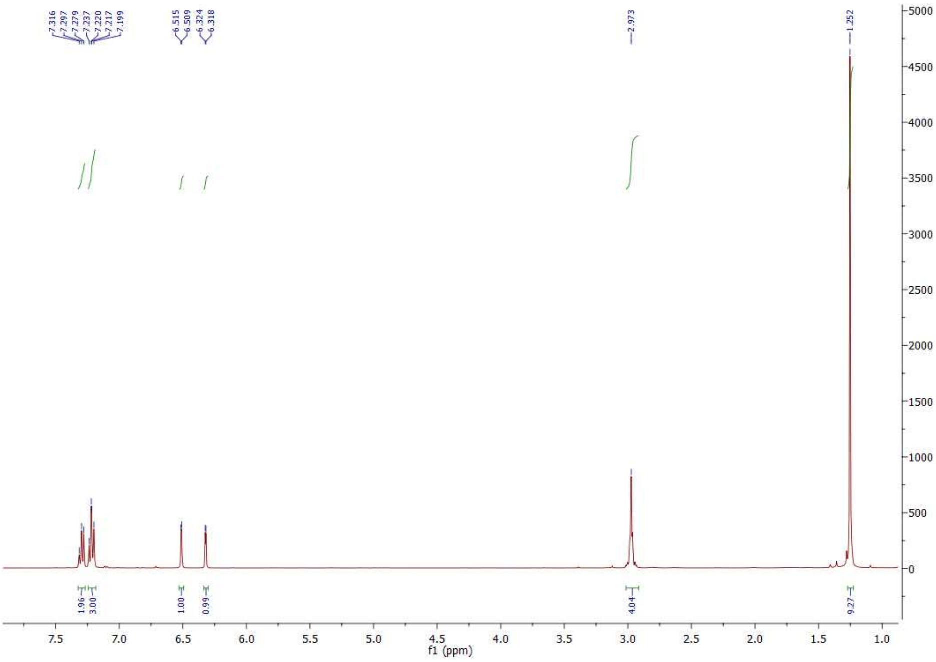

^{13}C NMR spectrum of 2-*tert*-butyl-6-(phenethylthio)-1,4-benzoquinone (**9**)

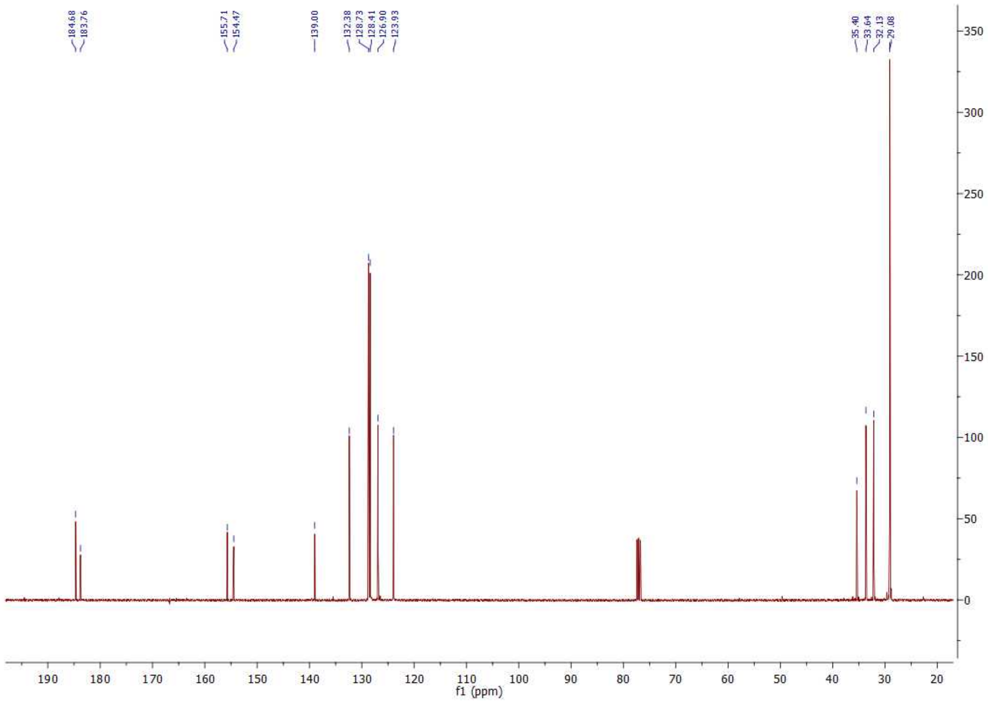

^{1}H NMR spectrum of 2-*tert*-butyl-6-(naphthalen-2-ylthio)-1,4-benzoquinone (**10**)

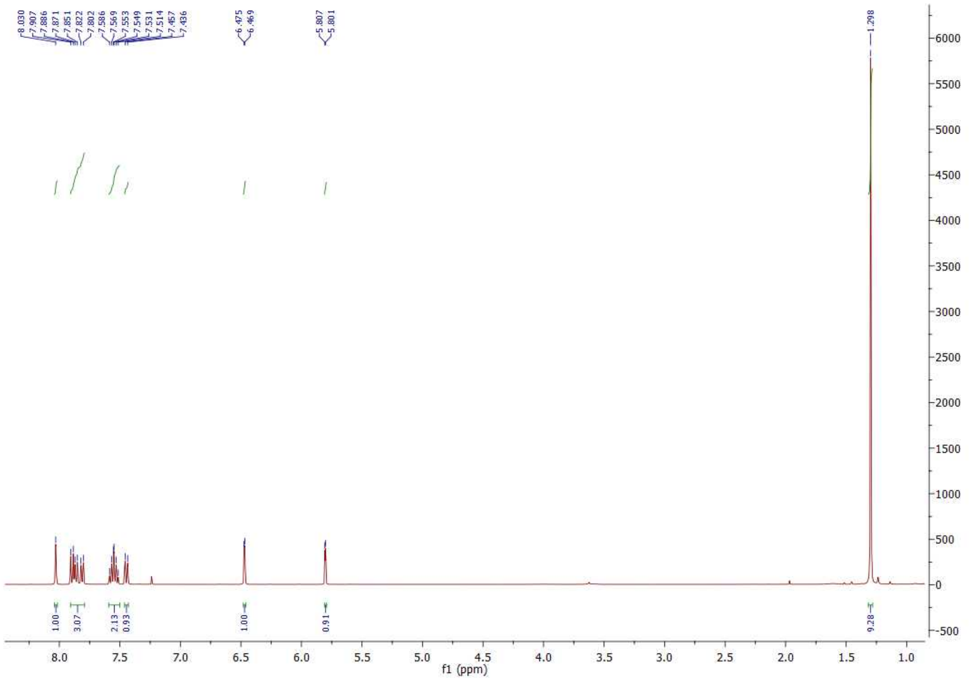

13C NMR spectrum of 2-*tert*-butyl-6-(naphthalen-2-ylthio)-1,4-benzoquinone (**10**)

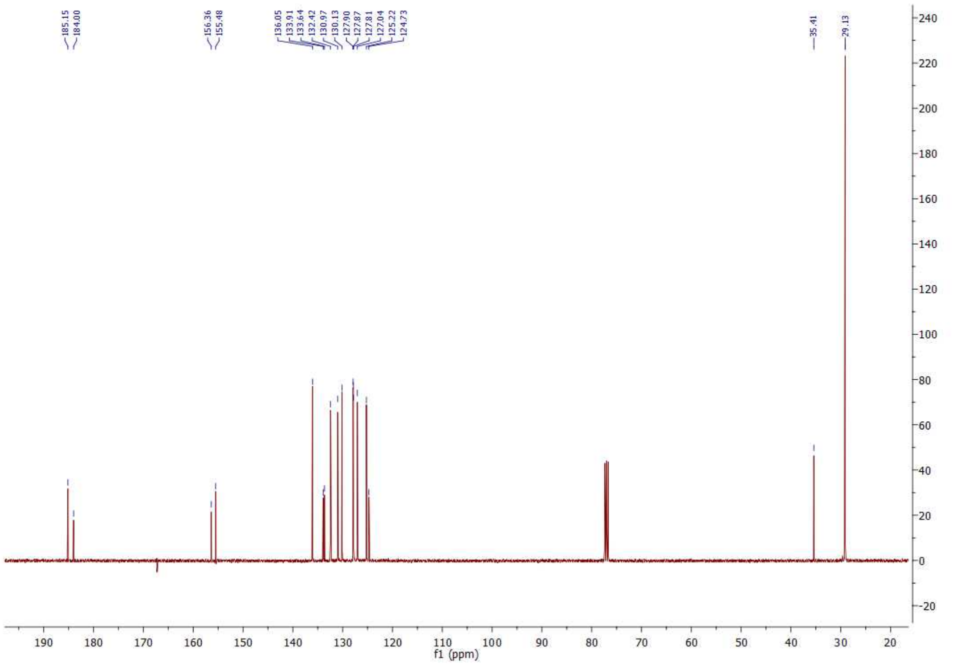

1.3 (+) ESI-MS spectra

(+) ESI-MS spectra um of compound **2**

Data File	INJG3_100V_4CE_FA_pos1.d	Sample Name	INJG3
Sample Type	Sample	Position	P2-B4
Instrument Name	6210QTOF	User Name	SYSTEM (SYSTEM)
Acq Method	Odredjivanje MM_100V_4CE_FA_pos.m	Acquired Time	14-Apr-25 13:58:20 (UTC+02:00)

Compound Table

Compound Label	RT	Mass	Abund	Formula	Tgt Mass	Diff (ppm)	Hits (DB)
Cpd 1: C12 H16 O3 S; 0.204	0.204	240.0831	11448	C12 H16 O3 S	240.082	4.36	1

Compound Label	*m/z*	RT	Algorithm	Mass
Cpd 1: C12 H16 O3 S; 0.204	241.0901	0.204	Find by Formula	240.0831

Compound Chromatograms

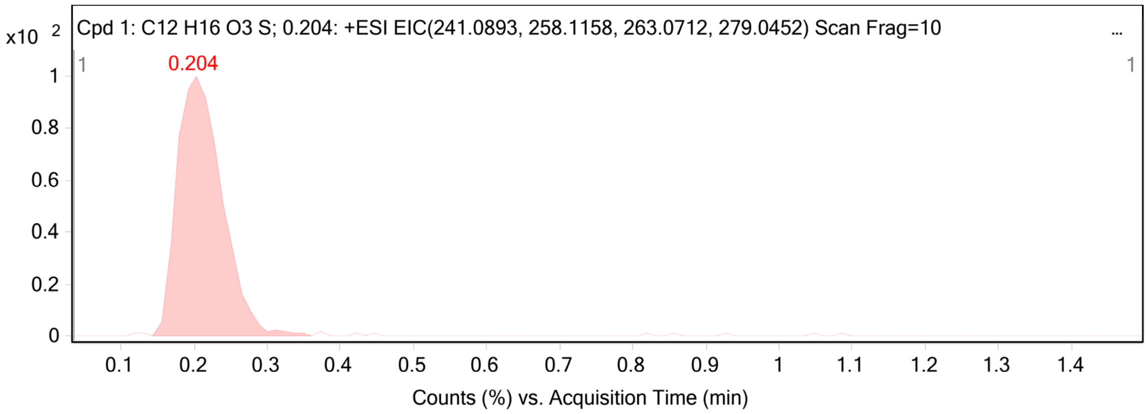

MS Zoomed Spectrum

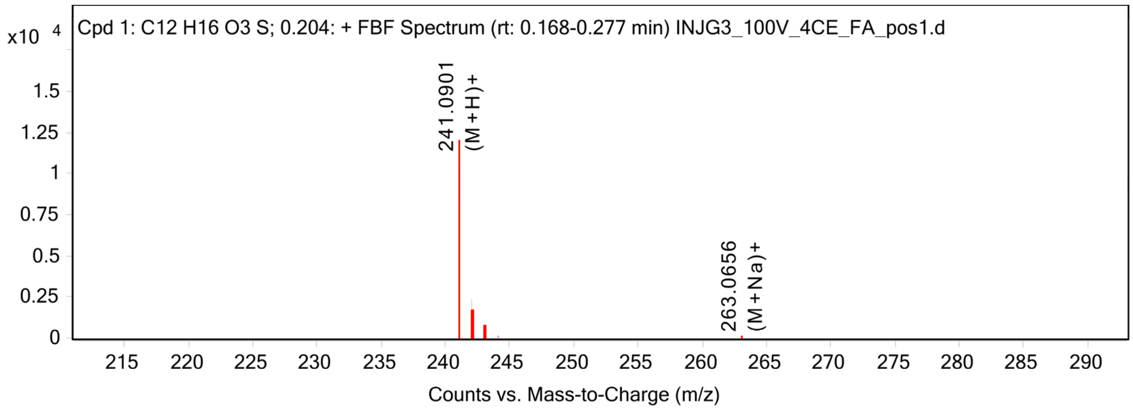

MS Spectrum Peak List

m/z	Calc *m/z*	Diff(ppm)	z	Abund	Ion
241.0901	241.0893	-3.41	1	11447.94	$(M+H)^+$
241.0901				11444.1	
242.0954	242.0925	-12.25	1	2308.4	$(M+H)^+$
243.0872	243.0876	1.49	1	685.23	$(M+H)^+$
263.0656	263.0712	21.29	1	134.12	$(M+Na)^+$

(+) ESI-MS spectrum of compound **3**

Data File	INJG4_100V_4CE_FA_pos1.d	Sample Name	INJG4
Sample Type	Sample	Position	P2-B5
Instrument Name	6210QTOF	User Name	SYSTEM (SYSTEM)
Acq Method	Odredjivanje MM_100V_4CE_FA_pos.m	Acquired Time	14-Apr-25 13:40:12 (UTC+02:00)

Compound Table

Compound Label	RT	Mass	Abund	Formula	Tgt Mass	Diff (ppm)	Hits (DB)
Cpd 1: C12 H16 O3 S; 0.207	0.207	240.0831	11453	C12 H16 O3 S	240.082	4.36	1

Compound Label	*m/z*	RT	Algorithm	Mass
Cpd 1: C12 H16 O3 S; 0.204	241.0905	0.207	Find by Formula	240.0831

Compound Chromatograms

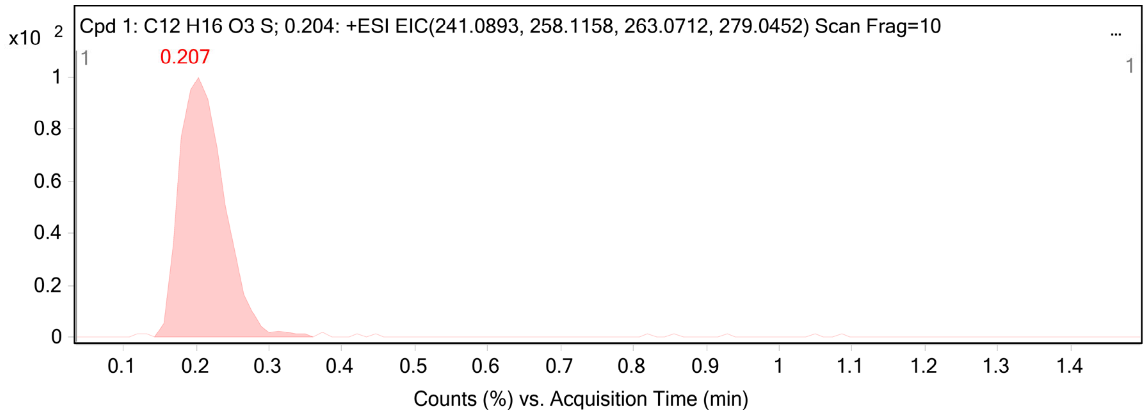

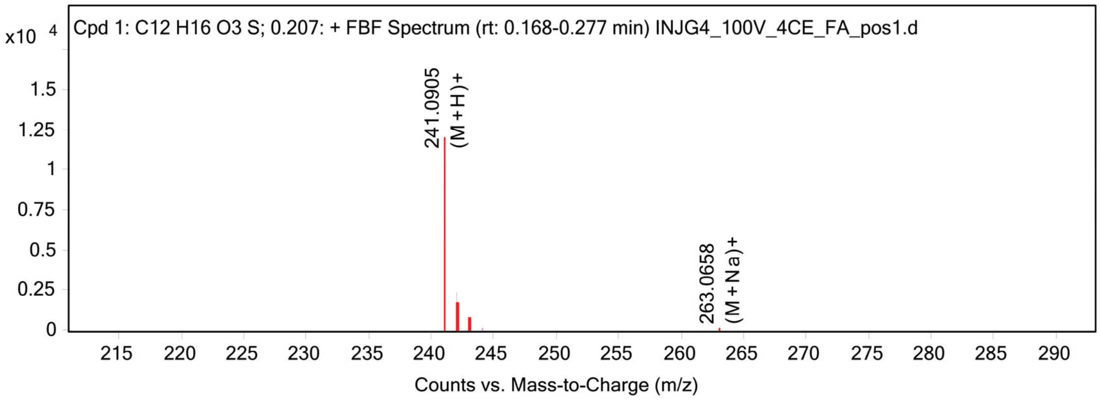

MS Spectrum Peak List

m/z	Calc *m/z*	Diff(ppm)	z	Abund	Ion
241.0905	241.0893	-3.97	1	11452.96	$(M+H)^+$
241.0905				11443.2	
242.0954	242.0925	-12.25	1	2312.6	$(M+H)^+$
243.0872	243.0876	1.49	1	688.32	$(M+H)^+$
263.0658	263.0712	21.29	1	135.18	(M+Na)+

(+) ESI-MS spectrum of compound **4**

Data File	VM020-2_100V_4CE_FA_pos1.d	Sample Name	VM020-2
Sample Type	Sample	Position	P2-B8
Instrument Name	6210QTOF	User Name	SYSTEM (SYSTEM)
Acq Method	Odredjivanje MM_100V_4CE_FA_pos.m	Acquired Time	14-Apr-25 14:08:10 (UTC+02:00)

Compound Table

Compound Label	RT	Mass	Abund	Formula	Tgt Mass	Diff (ppm)	Hits (DB)
Cpd 1: C15 H20 O2 S; 0.195	0.195	264.1206	195717	C15 H20 O2 S	264.1184	8.24	1

Compound Label	*m/z*	RT	Algorithm	Mass
Cpd 1: C15 H20 O2 S; 0.195	265.1276	0.195	Find by Formula	264.1206

Compound Chromatograms

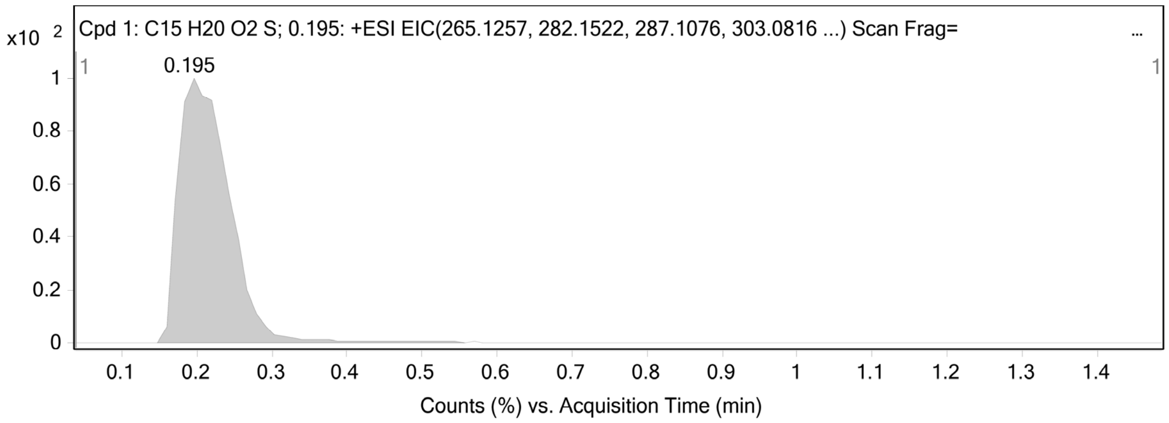

MS Zoomed Spectrum

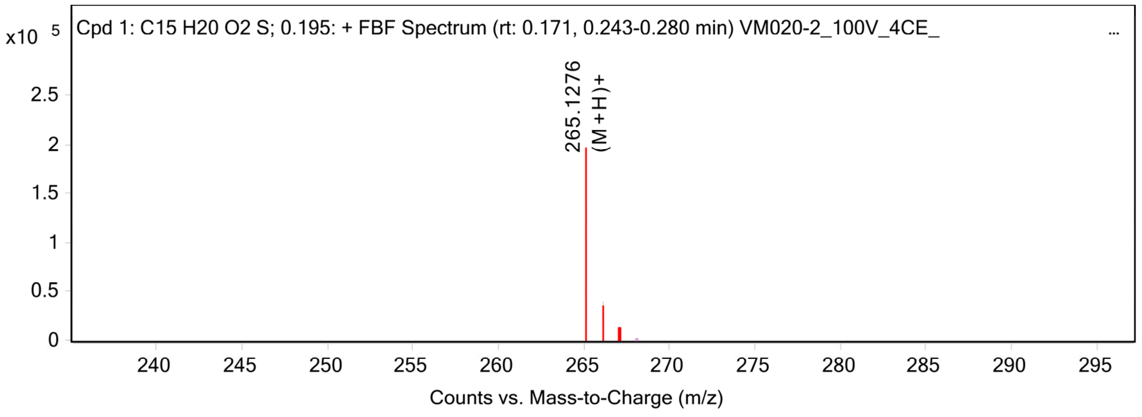

MS Spectrum Peak List

m/z	Calc *m/z*	Diff(ppm)	z	Abund	Ion
265.1276	265.1257	-7.34	1	195717.08	$(M+H)^+$
265.1276				195701.6	
266.1319	266.1289	-11.36	1	39287.52	$(M+H)^+$
267.128	267.1244	-13.6	1	8334.02	$(M+H)^+$

(+) ESI-MS spectrum of compound **5**

Data File	VM020-1_100V_4CE_FA_pos1.d	Sample Name	VM020-1
Sample Type	Sample	Position	P2-B7
Instrument Name	6210QTOF	User Name	SYSTEM (SYSTEM)
Acq Method	Odredjivanje MM_100V_4CE_FA_pos.m	Acquired Time	14-Apr-25 13:20:10 (UTC+02:00)

Compound Table

Compound Label	RT	Mass	Abund	Formula	Tgt Mass	Diff (ppm)	Hits (DB)
Cpd 1: C15 H20 O2 S; 0.195	0.197	264.1206	195725	C15 H20 O2 S	264.1184	8.24	1

Compound Label	*m/z*	RT	Algorithm	Mass
Cpd 1: C15 H20 O2 S; 0.195	265.1273	0.197	Find by Formula	264.1206

Compound Chromatograms

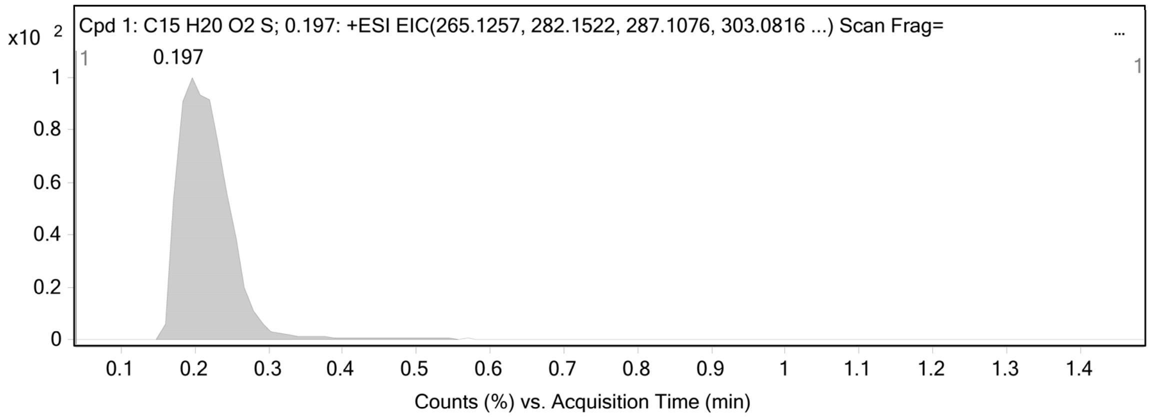

MS Zoomed Spectrum

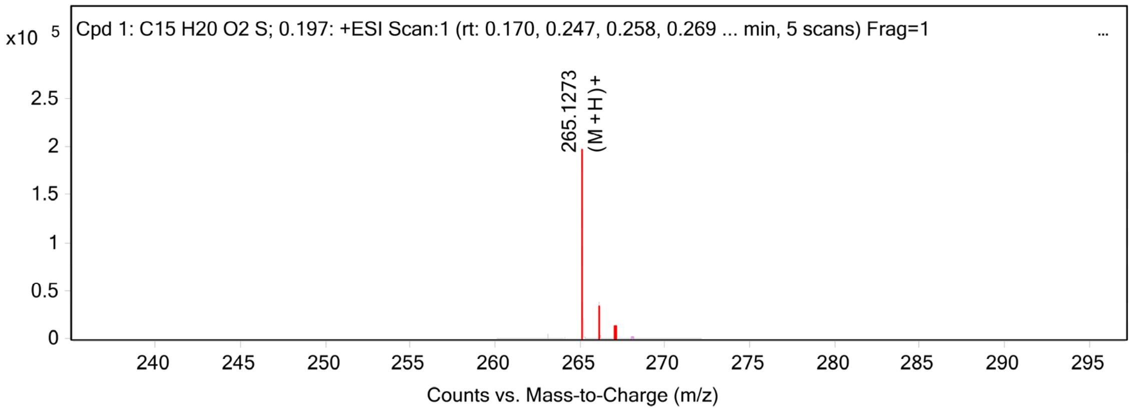

MS Spectrum Peak List

m/z	Calc *m/z*	Diff(ppm)	z	Abund	Ion
265.1273	265.1257	-6.03	1	195725.18	$(M+H)^+$
265.1276				195701.6	
266.1319	266.1289	-11.36	1	39296.78	$(M+H)^+$
267.128	267.1244	-13.6	1	8345.12	$(M+H)^+$

(+) ESI-MS spectrum of compound **6**

Data File	VM021-2_100V_4CE_FA_pos1.d	Sample Name	VM021-2
Sample Type	Sample	Position	P2-B10
Instrument Name	6210QTOF	User Name	SYSTEM (SYSTEM)
Acq Method	Odredjivanje MM_100V_4CE_FA_pos.m	Acquired Time	14-Apr-25 14:13:04 (UTC+02:00)

Compound Table

Compound Label	RT	Mass	Abund	Formula	Tgt Mass	Diff (ppm)	Hits (DB)
Cpd 1: C16 H16 O2 S; 0.193	0.193	272.0878	160265	C16 H16 O2 S	272.0871	2.45	1

Compound Label	*m/z*	RT	Algorithm	Mass
Cpd 1: C16 H16 O2 S; 0.193	273.0948	0.193	Find by Formula	272.0878

Compound Chromatograms

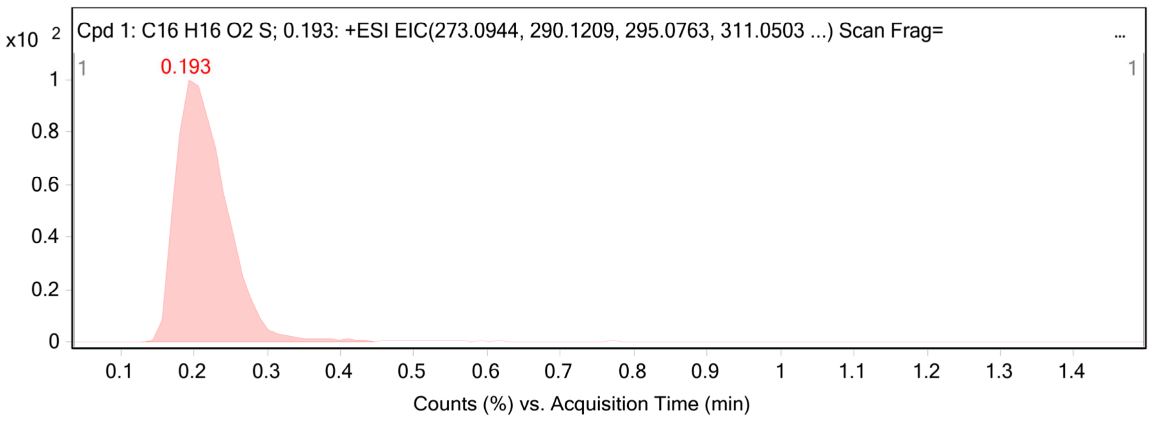

MS Zoomed Spectrum

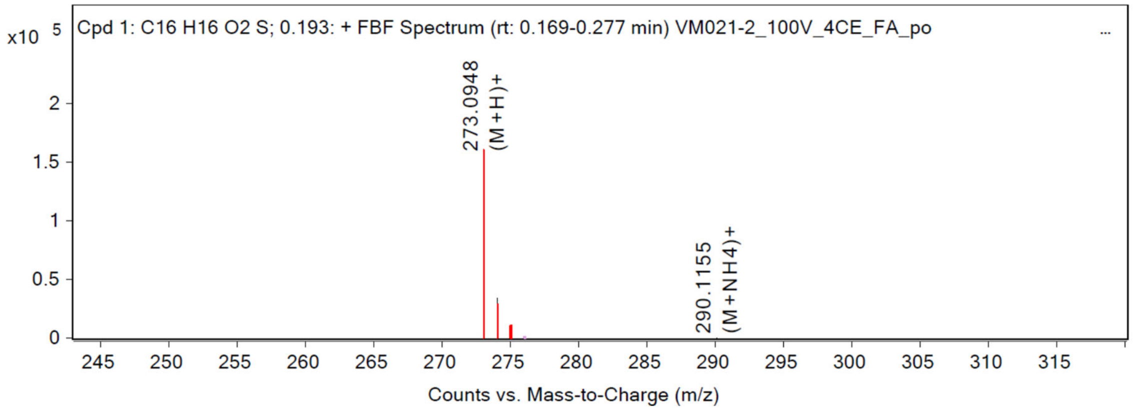

MS Spectrum Peak List

m/z	Calc *m/z*	Diff(ppm)	z	Abund	Ion
273.0948	273.0944	-1.52	1	160265.47	$(M+H)^+$
273.0948				160234.33	
274.099	274.0976	-5.27	1	34059.11	$(M+H)^+$
275.0961	275.0933	-10.08	1	6743.26	$(M+H)^+$
290.1155	290.1209	18.74	1	56.25	$(M+NH_4)^+$

(+) ESI-MS spectrum of compound **7**

Data File	INAK4_100V_4CE_FA_pos6.d	Sample Name	INAK4
Sample Type	Sample	Position	P2-C2
Instrument Name	6210QTOF	User Name	SYSTEM (SYSTEM)
Acq Method	Odredjivanje MM_100V_4CE_FA_pos.m	Acquired Time	14-Apr-25 15:12:04 (UTC+02:00)

Compound Table

Compound Label	RT	Mass	Abund	Formula	Tgt Mass	Diff (ppm)	Hits (DB)
Cpd 1: C16 H16 O3 S; 0.197	0.197	288.0821	94677	C16 H16 O3 S	288.082	0.39	1

Compound Label	*m/z*	RT	Algorithm	Mass
Cpd 1: C16 H16 O3 S; 0.197	289.0893	0.197	Find by Formula	288.0821

Compound Chromatograms

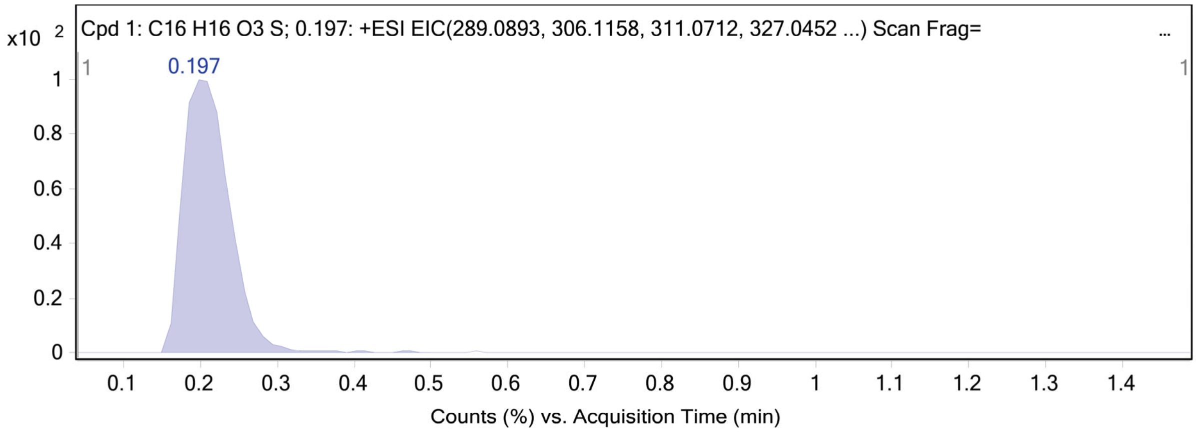

MS Zoomed Spectrum

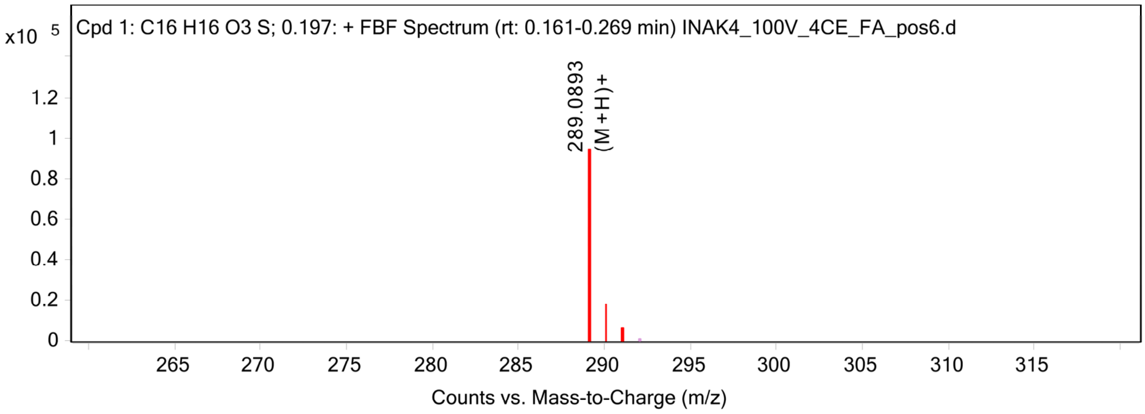

MS Spectrum Peak List

m/z	Calc *m/z*	Diff(ppm)	z	Abund	Ion
289.0893	289.0893	-0.09		94660.85	
289.0893	289.0893	-0.1	1	94676.7	$(M+H)^+$
290.0928	290.0925	-0.85	1	17939.79	$(M+H)^+$
291.0899	291.0884	-5.11	1	4093.77	$(M+H)^+$

(+) ESI-MS spectrum of compound **8**

Data File	INMTM1_100V_4CE_FA_pos2.d	Sample Name	INMTM1
Sample Type	Sample	Position	P2-B6
Instrument Name	6210QTOF	User Name	SYSTEM (SYSTEM)
Acq Method	Odredjivanje MM_100V_4CE_FA_pos.m	Acquired Time	14-Apr-25 14:42:12 (UTC+02:00)

Compound Table

Compound Label	RT	Mass	Abund	Formula	Tgt Mass	Diff (ppm)	Hits (DB)
Cpd 1: C17 H18 O3 S; 0.203	0.203	302.0965	196247	C17 H18 O3 S	302.0977	-3.72	1

Compound Label	*m/z*	RT	Algorithm	Mass
Cpd 1: C17 H18 O3 S; 0.203	303.1036	0.203	Find by Formula	302.0965

Compound Chromatograms

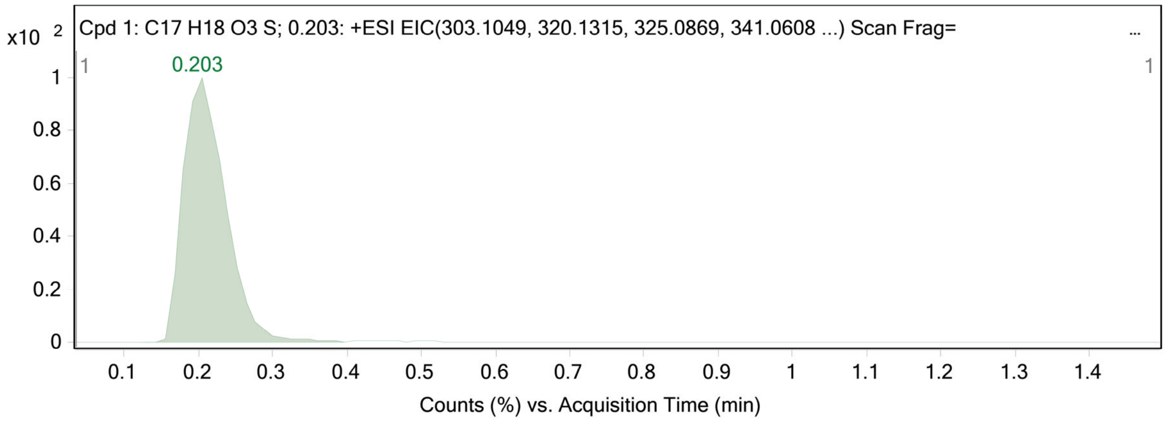

MS Zoomed Spectrum

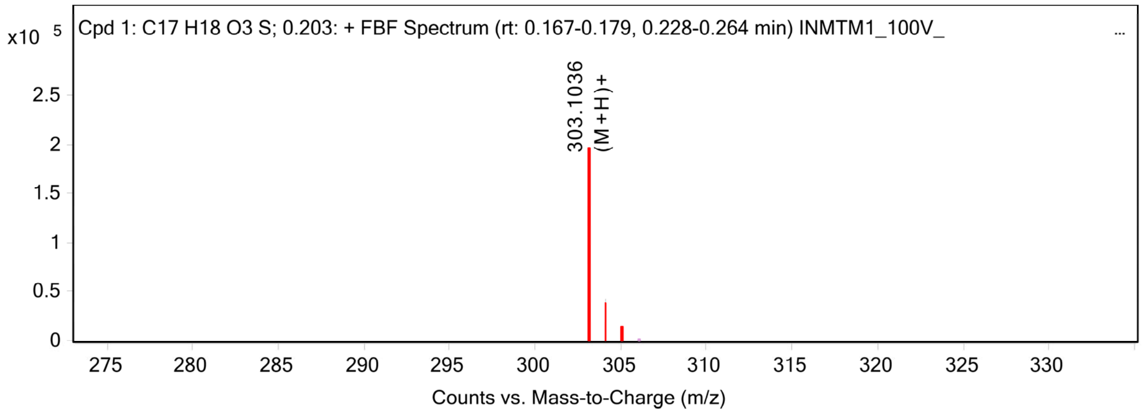

MS Spectrum Peak List

m/z	Calc *m/z*	Diff(ppm)	z	Abund	Ion
303.1036	303.1049	4.4	1	196247.3	$(M+H)^+$
303.1036				196219.11	
304.1076	304.1082	1.77	1	42679.73	$(M+H)^+$
305.1049	305.1043	-1.97	1	9432.11	$(M+H)^+$

(+) ESI-MS spectrum of compound **9**

Data File	INAK1_100V_4CE_FA_pos1.d	Sample Name	INAK1
Sample Type	Sample	Position	P2-B11
Instrument Name	6210QTOF	User Name	SYSTEM (SYSTEM)
Acq Method	Odredjivanje MM_100V_4CE_FA_pos.m	Acquired Time	14-Apr-25 14:15:31 (UTC+02:00)

Compound Table

Compound Label	RT	Mass	Abund	Formula	Tgt Mass	Diff (ppm)	Hits (DB)
Cpd 1: C18 H20 O2 S; 0.208	0.208	300.1188	123064	C18 H20 O2 S	300.1184	1.44	1

Compound Label	*m/z*	RT	Algorithm	Mass
Cpd 1: C18 H20 O2 S; 0.208	301.1258	0.208	Find by Formula	300.1188

Compound Chromatograms

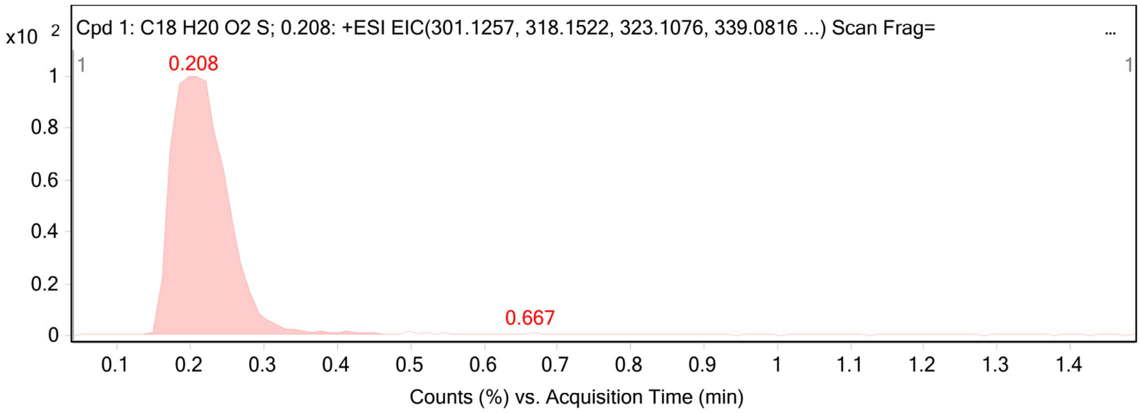

MS Zoomed Spectrum

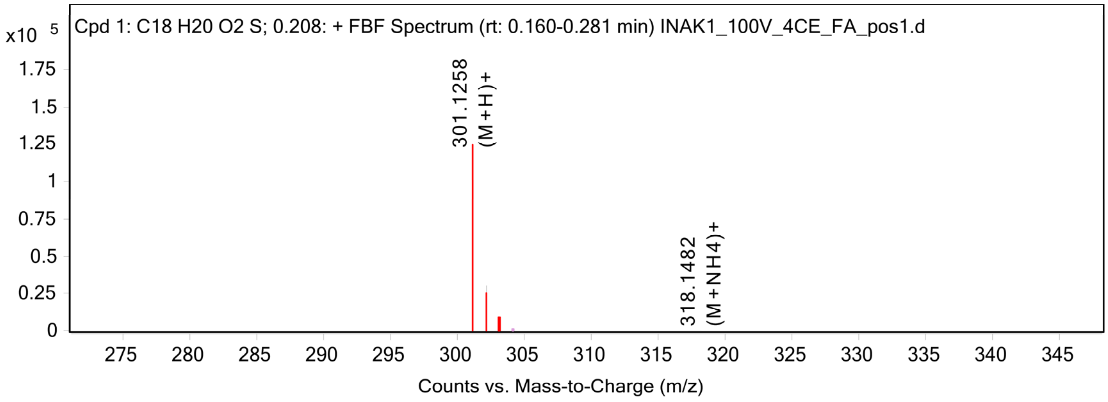

MS Spectrum Peak List

m/z	Calc *m/z*	Diff(ppm)	z	Abund	Ion
301.1258	301.1257	-0.46	1	123064.45	$(M+H)^+$
301.1258				122992.8	
302.1301	302.1289	-4.03	1	30101.46	$(M+H)^+$
303.1277	303.1251	-8.62	1	5853.41	$(M+H)^+$
318.1482	318.1522	12.59	1	82.23	$(M+NH_4)^+$

(+) ESI-MS spectrum of compound **10**

Data File	INAK3_100V_4CE_FA_pos2.d	Sample Name	INAK3
Sample Type	Sample	Position	P2-C1
Instrument Name	6210QTOF	User Name	SYSTEM (SYSTEM)
Acq Method	Odredjivanje MM_100V_4CE_FA_pos.m	Acquired Time	14-Apr-25 14:44:39 (UTC+02:00)

Compound Table

Compound Label	RT	Mass	Abund	Formula	Tgt Mass	Diff (ppm)	Hits (DB)
Cpd 1: C20 H18 O2 S; 0.205	0.205	322.1026	172006	C20 H18 O2 S	322.1028	-0.41	1

Compound Label	*m/z*	RT	Algorithm	Mass
Cpd 1: C20 H18 O2 S; 0.205	323.1096	0.205	Find by Formula	322.1026

Compound Chromatograms

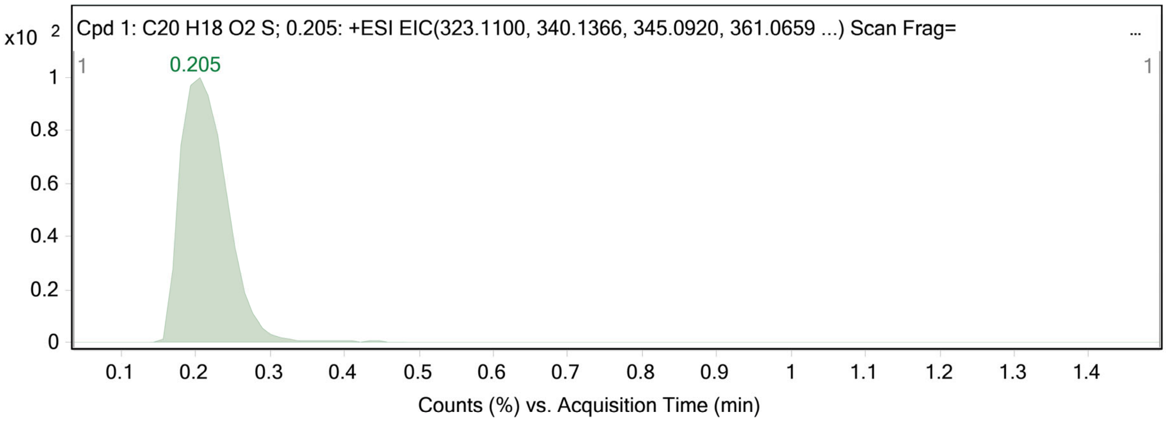

MS Zoomed Spectrum

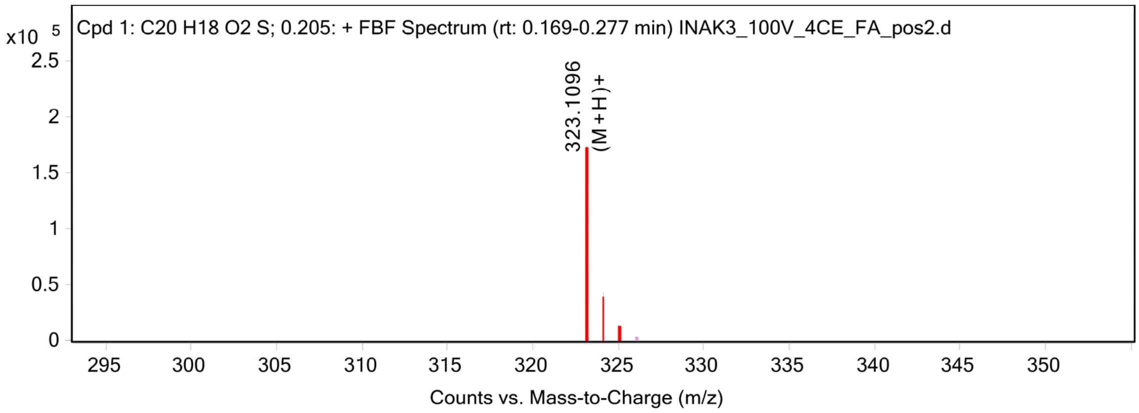

MS Spectrum Peak List

m/z	Calc *m/z*	Diff(ppm)	z	Abund	Ion
323.1096	323.11	1.18		171945.71	
323.1096	323.11	1.18	1	172005.63	$(M+H)^+$
324.1137	324.1133	-1.28	1	43456.55	$(M+H)^+$
325.1119	325.1099	-6.16	1	9073.24	$(M+H)^+$

2. Representative plots of three modes of inhibition

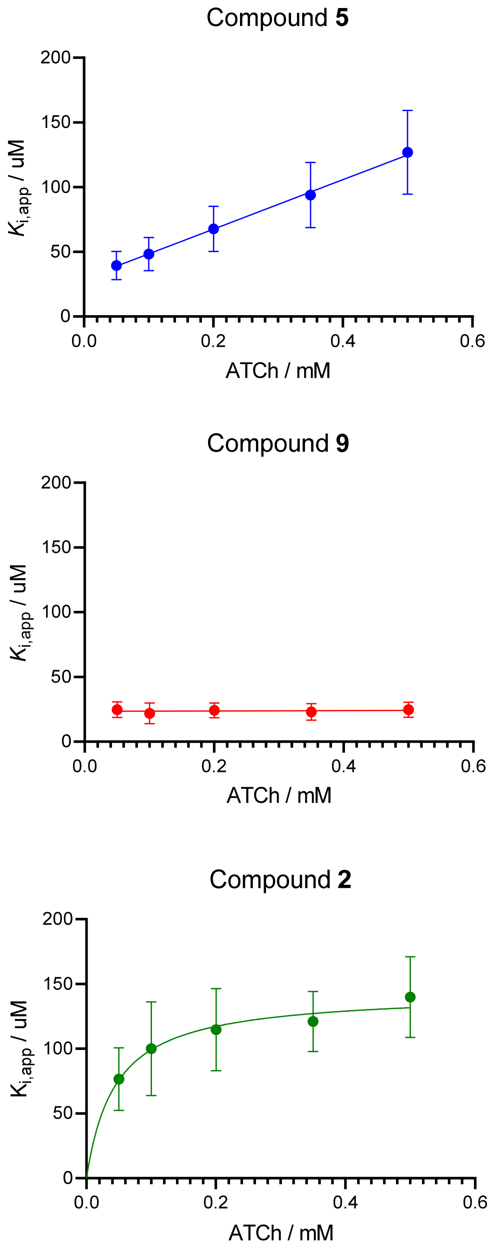

Figure S1. Representative plots of competitive (compound **5**), non-competitive (compound **9**) and mixed (compound **2**) type of inhibition of AChE.

2. 2D view of interactions

2.1. AChE

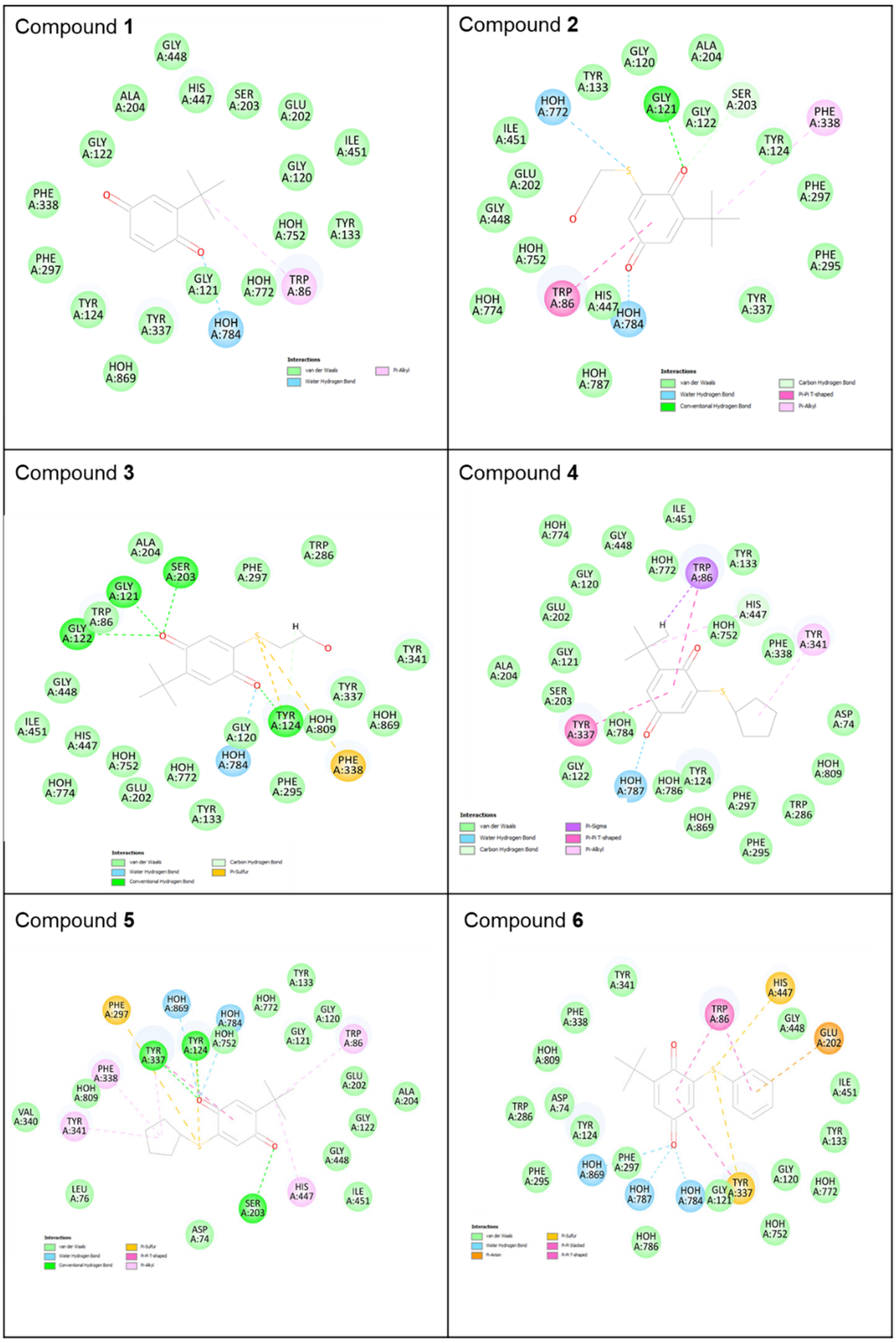

Figure S2. 2D view of interactions of modeled complex of AChE and compounds **1** – **6**. Dashed lines represent different types of non-bonding intermolecular interactions (magenta - π-alkyl, π-π interaction, orange - electrostatic interaction, green - conventional hydrogen bond, light green - carbon hydrogen bond, blue - water hydrogen bond). Red spheres represent conserved water molecules; only water molecules predicted to interact with ligands are shown.

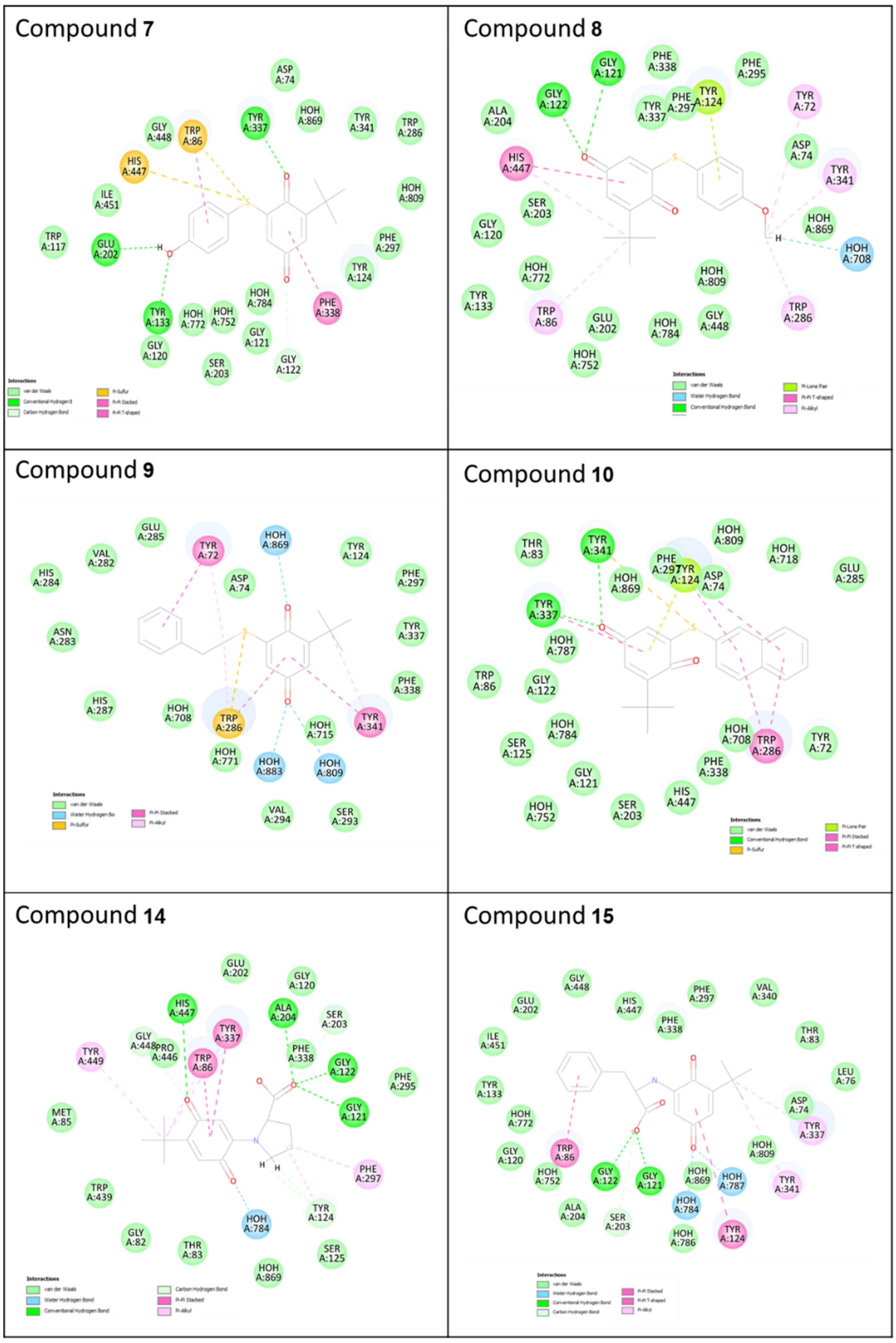

Figure S3. 2D view of interactions of modeled complex of AChE and compounds **7 – 10, 14** and **15**. Dashed lines represent different types of non-bonding intermolecular interactions (magenta - π-alkyl, π-π interaction, orange - electrostatic interaction, green - conventional hydrogen bond, light green - carbon hydrogen bond, blue - water hydrogen bond). Red spheres represent conserved water molecules; only water molecules predicted to interact with ligands are shown.

2.1. BChE and selected ligands

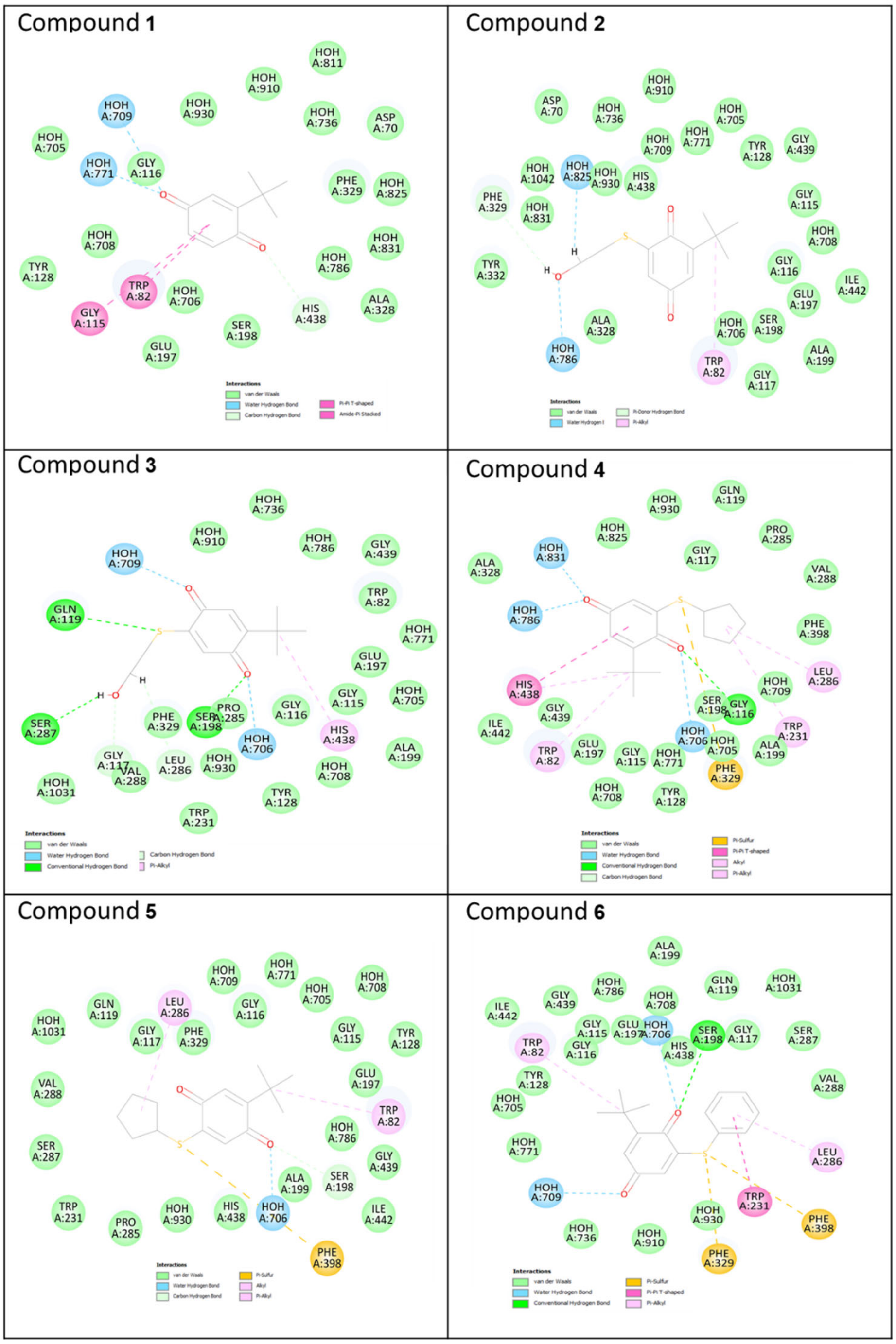

Figure S4. 2D view of interactions of modeled complex of BChE and compounds **1** – **6**. Dashed lines represent different types of non-bonding intermolecular interactions (magenta - π-alkyl, π-π interaction, orange - electrostatic interaction, green - conventional hydrogen bond, light green - carbon hydrogen bond, blue - water hydrogen bond). Red spheres represent conserved water molecules; only water molecules predicted to interact with ligands are shown.

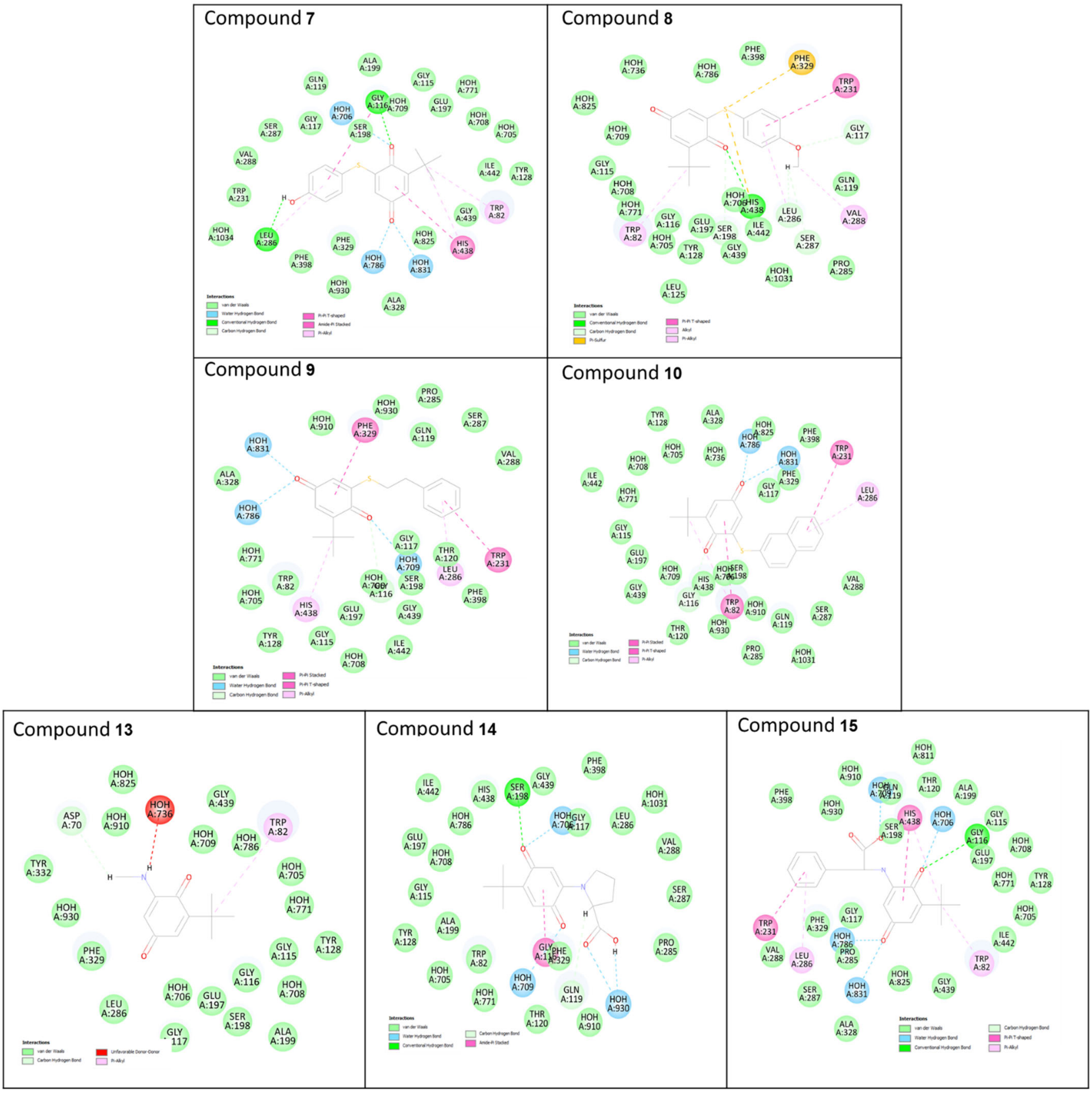

Figure S5. 2D view of interactions of modeled complex of BChE and compounds **7 – 10**, **12, 14** and **15**. Dashed lines represent different types of non-bonding intermolecular interactions (magenta - π-alkyl, π-π interaction, orange - electrostatic interaction, green - conventional hydrogen bond, light green - carbon hydrogen bond, blue - water hydrogen bond). Red spheres represent conserved water molecules; only water molecules predicted to interact with ligands are shown.

3. Calculated physical-chemical parameters of tested 1,4-benzoquinones

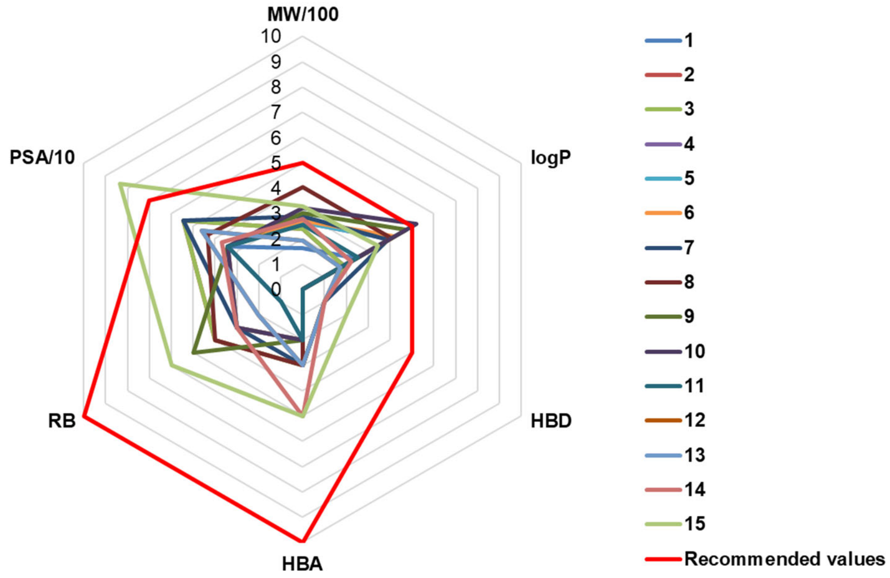

Figure S6. Radar plot of physicochemical properties (molecular weight, MW; lipophilicity coefficient, logP; number of hydrogen bonds donors, HBD, and acceptors HBA; rotatable bonds, RB; polar surface area, PSA) of the tested 1,4-benzoquinones. The recommended values for the CNS-active drugs are presented by a dashed red line.

4. Human intestinal absorption

Table S1. *In silico* estimated % of compounds that will be absorbed through the human intestine.

Compound	Human intestinal absorption %
**1**	96.9
**2**	94.9
**3**	94.0
**4**	95.1
**5**	93.7
6	98.5
**7**	92.2
**8**	96.4
**9**	95.0
**10**	94.2
**11**	94.7
**12**	88.4
**13**	88.4
**14**	93.1
**15**	93.3

References

30. Jeremić, M.; Pešić, M.; Dinić, J.; Banković, J.; Novaković, I.; Šegan, D.; Sladić, D. Simple avarone mimetics as selective agents against multidrug resistant cancer cells. *Eur. J. Med. Chem.* **2016**, *118*, 107–120. https://doi.org/10.1016/j.ejmech.2016.04.011.

31. Božić, T.; Novaković, I.; Gašić, M.J.; Juranić, Z.; Stanojković, T.; Tufegdžić, S.; Kljajić, Z.; Sladić, D. Synthesis and biological activity of derivatives of the marine quinone avarone. *Eur. J. Med. Chem.* **2010**, *45*, 923–929. https://doi.org/10.1016/j.ejmech.2009.11.0331.

32. Vilipić, J.; Novaković, I.; Stanojković, T.; Matić, I.; Šegan, D.; Sladić, D. Synthesis and biological activity of amino acid derivatives of avarone and its model compound. *Bioorg. Med. Chem.* **2015**, *23*, 6930–6942. https://doi.org/10.1016/j.bmc.2015.09.044.

33. Vilipić, P.; Novaković, I.T.; Zlatović, M.V.; Vujčić, M.T.; Tufegdžić, S.J.; Sladić, D.M. Interactions of cytotoxic amino acid derivatives of tert-butylquinone with DNA and lysozyme. *J. Serb. Chem. Soc.* **2016**, *81*, 1345–1358. https://doi.org/10.2298/JSC160725101V.

63. Jeremić, M.; Dinić, J.; Pešić, M.; Stepanović, M.; Novaković, I.; Šegan, D.; Sladić D. Alkylamino and aralkylamino derivatives of avarone and its mimetic as selective agents against non-small cell lung cancer cells, their antibacterial and antifungal potential. *J. Serb. Chem. Soc.,* **2018**, 83, 1193−1207; https://doi.org/10.2298/JSC180627062J.
